# Cryptic species within cryptic moths: new species of *Dunama* Schaus (Notodontidae, Nystaleinae) in Costa Rica

**DOI:** 10.3897/zookeys.264.4440

**Published:** 2013-02-06

**Authors:** Isidro A. Chacón, Daniel H. Janzen, Winnie Hallwachs, J. Bolling Sullivan, Mehrdad Hajibabaei

**Affiliations:** 1 Instituto Nacional de Biodiversidad (INBio), Apdo. 22-3100, Sto. Domingo, Heredia, Costa Rica; 2 Department of Biology, University of Pennsylvania, Philadelphia, PA 19104, USA; 3 200 Craven St., Beaufort, North Carolina 28516; 4 Department of Integrative Biology, University of Guelph, Guelph, Ontario, Canada N1G2W1

**Keywords:** *Dunama*, *Heliconia*, *Musa*, Arecaceae, caterpillars, moths, inventory, DNA barcodes, tropical forest, Area de Conservacion Guanacaste, INBio

## Abstract

Based on almost 1,700 recently reared and wild-collected specimens, the genus *Dunama* Schaus (Notodontidae, Nystaelinae) in Costa Rica is reviewed. Eight species are recorded of which seven are newly described: *Dunama jessiehillae* Chacón, *Dunama jessiebarronae* Chacón, *Dunama janewaldronae* Chacón, *Dunama jessiebancroftae* Chacón, *Dunama janecoxae* Chacón, *Dunama biosise* Chacón, *Dunama indereci* Chacón. *Dunama angulinea* Schaus is redescribed and associated with its correct genitalia. *Dunama tuna* (Schaus), previously listed as ocurring in Costa Rica, is restricted to Colombia. Most species are described through their distinctive CO1 barcodes, genitalia and life histories. *Dunama* adults and caterpillars, their foodplants, and their parasites in Area de Conservación Guanacaste (ACG) in northwestern Costa Rica are described where known. Many life history stages are illustrated.

## Introduction

Schaus (1912) established the genus *Dunama* for a group of small, relatively drab, mottled and tree-bark patterned, brown notodontid moths with a black orbicular spot. Todd (1976) revised the genus whose distribution extends from Mexico to Amazonian Brazil. He described two new species and listed two species, *Dunama angulinea* Schaus and *Dunama tuna* (Schaus), from Costa Rica. One additional species was recently described by Miller and Thiaucourt (2011) from Ecuador. The genus traditionally has been placed in the Nystaleinae, but that placement remains provisional because species of *Dunama* lack the characteristic morphological traits of most nystaleines. Additionally, all known caterpillars of *Dunama* feed on monocots (Musaceae, Marantaceae, Heliconiaceae, Arecaceae), a trait rarely encountered in the Notodontidae. Review of the Costa Rican species is part of an ongoing documentation of over 700 notodontid species collected or reared by parataxonomists and others in Area de Conservación Guanacaste (Janzen 2004, Janzen et al. 2009, Janzen and Hallwachs 2011, 2012, and see http://janzen.bio.upenn.edu/caterpillars/database.lasso ).

## Material and methods

About 1,700 spread specimens of *Dunama* spp. were examined as follows: 1,545 rearing records from the project “Inventory of the caterpillars of Area de Conservación Guanacaste (ACG), and their parasitoids and food plants” (see Janzen 2004, Janzen et al. 2009, Janzen and Hallwachs 2011, 2012 and also search on *Dunama* spp. at http://janzen.bio.upenn.edu/caterpillars/database.lasso ), where the species reside under their interim names until this paper is published. 73 light-caught specimens from the INBio Lepidoptera collection of the project “National Inventory of Biodiversity 1978–2011.” 53 light-caught specimens from the collection of J. Bolling Sullivan, Beaufort, NC, USA.

Genital dissections and measurements were made using an Olympus SZ60 stereomicroscope with a calibrated ocular micrometer. The following protocol was used for the dissection of genitalia: abdomens were digested in 10% KOH, cleared, and stained with mercurochrome and Eosin Y (Montero-Ramírez et al. 2011). Genitalia and pelts were stored in glycerol for examination (in 70% ethanol solution, 3:1) and subsequently slide mounted using Euparal. Genital slides were photographed using a JVC 3-CCD color video camera attached to an Olympus SZ60 stereomicroscope, both mounted in an Olympus SZH-ILLD illumination base. Using Montage explorer software (version: 2.01.0075, Synoptics Ltd.) and Auto-Montage software (version: 4.02.0014, Synoptics Ltd.) photographs were enhanced for publication. Morphological terminology follows Miller (1991). A subset of the total specimens was used for species descriptions.

All holotypes and representative paratypes are deposited in the collections at the Instituto Nacional de Biodiversidad (INBio), Santo Domingo de Heredia, Costa Rica, and the other paratypes are in the USNM.

### Repository abbreviations

**INBio **Instituto Nacional de Biodiversidad, Santo Domingo de Heredia, Costa Rica

**JBS **J. Bolling Sullivan, Beaufort, North Carolina, USA

**USNM **National Museum of Natural History, Smithsonian Institution, Washington, District of Columbia, USA

### Key to morphological terminology

**WL** Wing length

**AD** Adterminal line

**M** Medial line

**PM** Postmedial line

**ST 8** Sternum 8

**T8** Tergum 8

**CB** Corpus bursae

**DB** Ductus bursae

## Systematics

### Dunama

Schaus

http://species-id.net/wiki/Dunama

Dunama Schaus, 1912: 52.

#### Type species.

*Dunama angulinea* Schaus, 1912: 52; Draudt 1932: 981; Gaede 1934: 263; Todd 1976: 190–192.

#### Diagnosis.

***Adults*** – Small to medium-sized notodontid moths, forewing 10–22 mm, females larger than males; male antenna bipectinate for 4/5 of length with pectinations decreasing toward antennal tip, last 1/5 simple; female antenna simple; palpi upcurved to medial area of frons, second segment 2 x first segment in length, 3^rd^ segment very small and slightly decumbent; scaling appressed; haustellum present, ocelli absent. Thoracic scaling not tightly appressed, without tufts, concolorous with forewing; abdominal scaling appressed, without tufts, concolorous with hindwing. Forewing with M1 from proximal third of narrow accessory cell; hindwing with Sc from middle of cell diverging from Rs and straight; Rs and M1 connate from upper angle and M3 and Cu1 connate from lower angle of the cell. Male terminal tergites distinctive. ***Male genitalia*** – Uncus short, rounded, sometimes divided; socii sclerotized, upcurved; valves with costal margin sclerotized, sometimes with projections; anal margin often partially but narrowly sclerotized and usually diagnostic; juxta undifferentiated, transtilla membraneous; phallus well developed, sclerotized, usually extending to uncus and narrowing distally; often with distinct lateral and dorsolateral processes; eighth sternum diagnostic, quadrate basally but often with multiple distal projections. ***Female genitalia*** – Ovipositor lobes large, often sclerotized, ostia sclerotized, ductus and corpus bursae reduced, membranous and without signa. ***Larvae*** – Brightly colored, feeding on monocots.

### Dunama angulinea

Schaus, 1912

http://species-id.net/wiki/Dunama_angulinea

Figs 1–6

#### Type material.

**Holotype** male: Guapiles, Costa Rica, 17505 USNM (examined).

#### Other material examined.

♂ Costa Rica, Limon Prov., Hitoy Cerere Reserve, 350 m, 9.404 N, -83.015 W, 1–4 July 2008, J. B. Sullivan (dissected); 3 ♂ Costa Rica, Limon Prov., Verugua Rainforest, 450 m, 9.653 N, -83.113 W, 12–16 March 2010, J. B. Sullivan (1 dissected, 3 barcoded).

#### Diagnosis.

Sternum 8 (St8) wide, short, anterior margin simple, posterior margin bearing a pair of small, widely-separated processes. Phallus thin basally, wider medially, with a pair of short, serrate projections on each margin, distal part with a pair of opposite, marginal, non-serrate projections, longer than anterior ones. Vesica short, unsclerotized, without cornuti. The single pair of terminal, widely-separated processes of sternum 8 and the tripartite distal structure of the phallus distinguishes *Dunama angulinea* from its known congeners.

#### Redescription.

**Figures 1–6.**
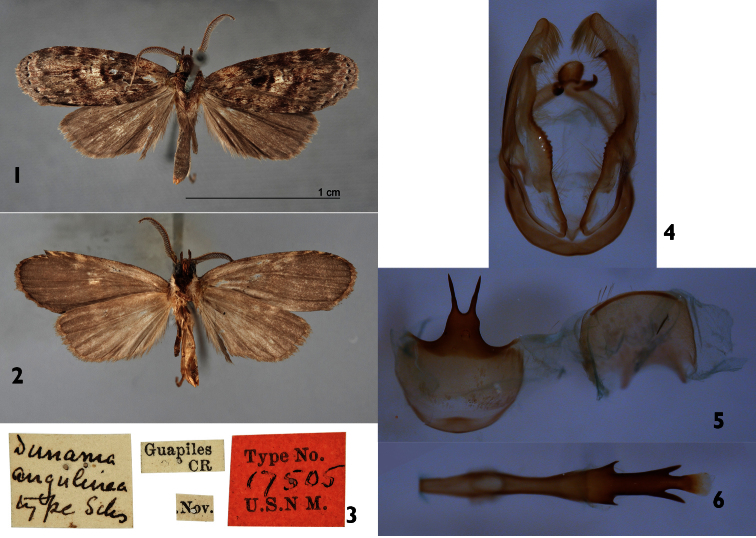
*Dunama angulinea* **1, 2** Male Holotype dorsal and ventral Type # 17505 USNM **3** Male Holotype labels **4** Male genitalia **5** Male St8 **6** Phallus.

**Male** (Figs 1–6). ***Head*** – Antenna pectinate in basal 4/5, rami moderately long, reddish brown, distal fifth of shaft simple, cream colored with an intermix of reddish-brown and gray-brown scales; scape with scale tuft reddish brown and cream colored; ocelli absent; labial palpus upcurved, reddish brown with a few scattered cream-colored scales; vertex reddish brown, cream colored laterally; patagium reddish brown near midline, reddish brown laterally, margins cream colored. ***Thorax and abdomen*** – Tegula cream colored at base, a mix of cream and reddish-brown scales distally; mesoscutum reddish brown anteriorly, cream and reddish brown posteriorly; mesoscutellum mostly creamy white; thoracic pleuron cream colored to reddish brown; legs mostly reddish brown on outer surfaces, cream colored on inner ones. Abdominal dorsum light gray, venter cream colored. ***Wings*** – Forewing dorsal ground color a mixture of gray-brown, reddish-brown, and beige-colored scales; veins lined with gray, especially distally; anal fold and cubitus reddish brown; orbicular spot diffuse reddish brown; reniform spot small, reddish brown; medial (M) line thin, reddish brown, a wide, vaguely-defined beige band beyond it; postmedial (PM) line thin, reddish brown, poorly defined; adterminal (AD) line reddish brown, fringe gray brown. Ventral surfaces of both wings gray brown (Figs 1, 2). Dorsal hindwing dirty gray brown, lighter near base. Wing length (WL) 11.20–12.2 mm). ***Male genitalia*** (Figs 4–6) – tergum 8 quadrate, posterior margin narrowly sclerotized; St8 wide, short, anterior margin simple, posterior margin extended and bearing a pair of long processes (Fig. 5). Uncus lobule-like, short and pubescent; socci thin, short and slightly curved. Valva broad and membranous, with saccular margin serrate, inner surface with spine-like process, near apex (Fig. 4). Phallus thin basally, wider medially, with a pair of short, serrate projections on each margin, distal part with a pair of opposite marginal non-serrate projections longer than anterior ones. Vesica short, unsclerotized (Fig. 6). **Female.** Unknown.

#### Natural history.

Unknown

#### Distribution.

Adults of *Dunama angulinea* have been collected from Limon Province (Hitoy Cerere, Verugua Rainforest, Guapiles). The distribution follows the Caribbean coast and we know of no records farther inland (Fig. 85). Two specimens identical in size and maculation from La Selva (Heredia) were found on dissection to represent another species described below.

#### Remarks.

Todd (1976, Fig. 10) illustrated the genitalia of what he supposed was *Dunama angulinea* from two paratypes from Guatemala (USNM slides ELT 822 ♂, 855 ♂). The holotype from Guapiles, Costa Rica was not dissected. When we dissected the type, it was obvious that the Guatemalan paratypes represent another species. We are not describing the Guatemalan species as new because its genitalia are very similar to those of *Dunama tuna* and because we have no specimens from the area between Guatemala and Costa Rica. We also have no barcode or life history data for the Guatemalan species. The barcode NJ tree (Fig. 86) associates *Dunama angulinea* with *Dunama jessiehillae*, described below, from western and inland Costa Rica and can be distinguished from it only by the shape of the sternum. One haplotype represents all three specimens of *Dunama angulinea* that are recent enough for molecular analysis.

### Dunama jessiehillae

Chacón sp. n.

urn:lsid:zoobank.org:act:A2F983CB-4888-40BD-BE13-BFBD02302B4E

http://species-id.net/wiki/Dunama_jessiehillae

Figs 7–14 , 74, 79–81

#### Type material.

**Holotype** male:99-SRNP-4120 (Dissected,COI Barcoded),Costa Rica, Prov. Alajuela,Sector San Cristobal, Sendero Vivero 10.86739, -85.38744, 730 m, 29 January 1999, Gloria Sihezar (INBio). **Paratypes**: Male:07-SRNP-23691 (COI Barcoded), Costa Rica, Prov. Guanacaste, Sector del Oro, Rio Chon 11.04118, -85.44170, 320 m, 28 September 2007, Elieth Cantillano. Female: 04-SRNP-42836 (COI Barcoded), Costa Rica, Prov. Alajuela, Sector Rincon Rain Forest, Sendero Rincon 10.8962, -85.27769, 430 m, 16 December 2004, Jose Perez. Female: 99-SRNP-4126.. (COI Barcoded), Costa Rica, Prov. Alajuela, Sector San Cristobal, Sendero Vivero 10.86739, -85.38744, 730 m, 29 January 1999, Gloria Sihezar. Male: 99-SRNP-4116 (COI Barcoded), Costa Rica, Prov. Alajuela, Sector San Cristobal, Sendero Vivero 10.86739, -85.38744, 730 m, 1 February 1999, Gloria Sihezar. Female: 99-SRNP-4118 (COI Barcoded), Costa Rica, Prov. Alajuela, Sector San Cristobal, Sendero Vivero 10.86739, -85.38744, 730 m, 28 January 1999, Gloria Sihezar. Female: 07-SRNP-23698 (COI Barcoded), Costa Rica, Prov. Guanacaste, Sector del Oro, Rio Chon 11.04118, -85.44170, 320 m, 27 September 2007, Elieth Cantillano. Male: 08-SRNP-41651 (COI Barcoded), Costa Rica, Prov. Alajuela, Sector Rincon Rain Forest, Quebrada Escondida 10.89928, -85.27486, 420 m, 2 September 2008, Anabelle Cordoba. Male: 05-SRNP-43080 (Dissected, COI Barcoded), Costa Rica, Prov. Alajuela,Sector Rincon Rain Forest, Anonas 10.90528, -85.27882, 405 m, 2 November 2005, Jose Perez.

#### Other material examined.

Barcoded: 198 specimens that divided into four haplotypes with slight differences from the most common haplotype (163 specimens) of 0.13% or less; we do not consider these differences to be of species-level significance and many are due to slightly shorter barcode sequences. No specimens from Heredia Province were barcoded. Museum specimens: (45 specimens) 2♂ 3♀ Guanacaste, 20♂ 10♀ Alajuela, 10♂ Heredia. Dissections: 1♂ 2♀ Guanacaste, 2♂ 1♀ Alajuela, 2♂ Heredia. INBio, USNM, JBS. **Janzen & Hallwachs vouchers of reared specimens. **Male: 07-SRNP-23702 (COI Barcoded), Costa Rica, Prov. Guanacaste, Sector del Oro, Rio Chon 11.04118, -85.44170, 320 m, 28 September 2007, Elieth Cantillano. Male: 04-SRNP-42845 (COI Barcoded), Costa Rica, Prov. Alajuela, Sector San Cristobal, Sendero Vivero 10.86739, -85.38744, 730 m, 16 December 2004, Gloria Sihezar. Female: 05-SRNP-43079 (COI Barcoded), Costa Rica, Prov. Alajuela, Sector Rincon Rain Forest, Anonas 10.90528, -85.27882, 405 m, 2 November 2005, Jose Perez. Female: 07-SRNP-23690 (COI Barcoded), Costa Rica, Prov. Guanacaste, Sector del Oro, Rio Chon 11.04118, -85.44170, 320 m, 27 September 2007, Elieth Cantillano. Male: 04-SRNP-42845 (COI Barcoded), Costa Rica, Prov. Alajuela, Sector San Cristobal, Sendero Vivero 10.86739, -85.38744, 730 m, 1 February 1999, Gloria Sihezar (INBio). Female: 99-SRNP- 4118, Costa Rica, Prov. Alajuela, Sector San Cristobal, Sendero Vivero 10.86739, -85.38744, 730 m, 28 January 1999, Gloria Sihezar. **INBio specimens.** Male: INBIOCRI002582936 (Dissected), Costa Rica, Prov. Heredia, La Selva Biol. Sta., Puerto Viejo de Sarapiqui 10.431958 -840091, 40 m, February 1986, M.M. Chavarria, A. Chacon. Male: INB0004268497 (COI Barcoded), Costa Rica, Prov. Alajuela, San Ramon, Est. Biol. Villa Blanca 10.201361, -84.485101, 1115 m, October 2009, R. Rojas (reared). Male: INB0004251816 (COI Barcoded, Dissected), Costa Rica, Prov. Alajuela, San Ramon, Est. Biol. Villa Blanca 10.201361, -84.485101, 1115 m, October 2009, R. Rojas (reared). Female: INB0004268498 (COI Barcoded), Costa Rica, Prov. Alajuela, San Ramon, Est. Biol. Villa Blanca 10.201361, -84.485101, 1115 m, October 2009, R. Rojas (reared). Female: INB0004268499 (COI Barcoded), Costa Rica, Prov. Alajuela, San Ramon, Est. Biol. Villa Blanca 10.201361, -84.485101, 1115 m, October 2009, R. Rojas (Reared). Female: INB0004251817 (COI Barcoded), Costa Rica, Prov. Alajuela,San Ramon, Est. Biol. Villa Blanca 10.201361, -84.485101, 1115 m, October 2009, R. Rojas (reared).

#### Etymology.

This species is named in honor of Ms. Jessie Hill of Hawaii and Philadelphia, and great-great-grandaughter of Ms. Jessie Barron, and in emphatic recognition of Jessie Hill’s contribution to saving and inventorying the conserved ACG rain forest in which reside *Dunama jessiehillae* and four other new species of *Dunama* described in this report.

#### Diagnosis.

St8 wide, short, anterior margin simple, posterior margin bearing a pair of small, widely separated processes, a second long pair of processes arises between this more basal pair. Phallus thin basally, wider medially, with a pair of short, serrate projections on each margin, distal part with a pair of opposite, marginal, non-serrate projections, longer than anterior ones. Vesica short, unsclerotized, no cornuti. The tripartite distal structure of the phallus and the two pairs of processes on St8 distinguish *Dunama jessiehillae* from its known congeners.

#### Description.

**Figures 7–14.**
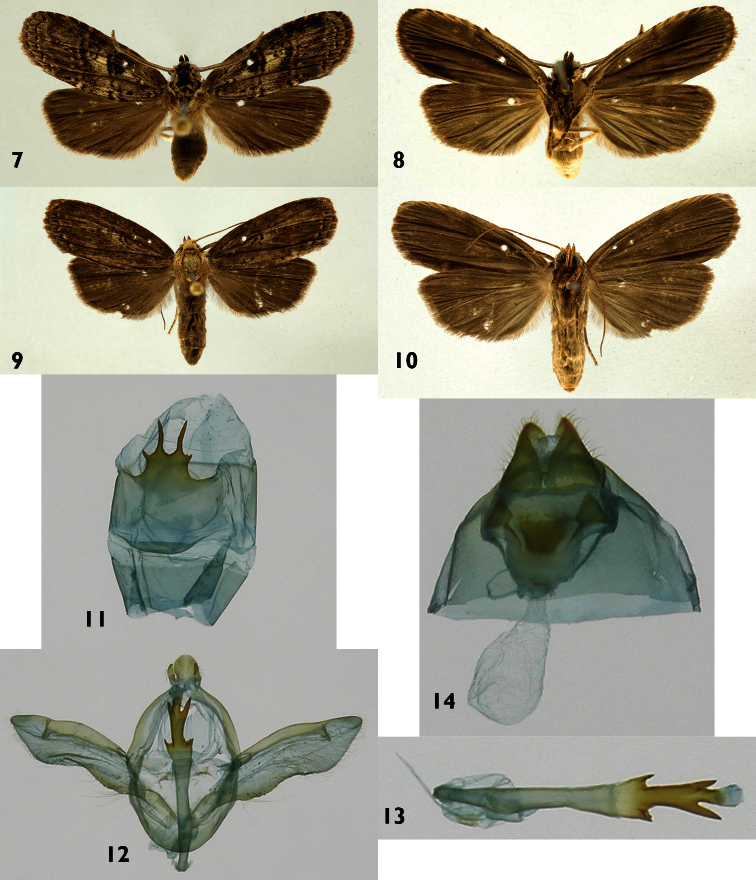
*Dunama jessiehillae* **7, 8** Male dorsal and ventral 99-SRNP-4120 **9, 10** Female dorsal and ventral 04-SRNP-42836 **11** Male St8 **12** Male genitalia99-SRNP-4120 **13** Phallus **14** Female genitalia04-SRNP-42836.

**Male** (Figs 7, 8, 11–13). ***Head*** – Antenna pectinate in basal 4/5, rami moderately long, reddish brown, distal fifth of shaft simple, cream colored with an intermix of reddish-brown and gray-brown scales; scape with scale tuft reddish brown and cream colored; frons with cream-colored scales interspersed with a few reddish-brown scales, ocelli absent; labial palpus upcurved, reddish brown with a few scattered cream-colored scales; vertex reddish brown, cream colored laterally; patagium reddish brown near the midline, reddish brown laterally, margins cream colored. ***Thorax and abdomen*** – Tegula cream colored at base, a mix of cream and reddish-brown scales distally; mesoscutum reddish brown anteriorly, cream and reddish brown posteriorly; mesoscutellum mostly creamy white; thoracic pleuron cream colored to reddish brown; legs mostly reddish brown on outer surfaces, cream colored on inner surfaces. Abdominal dorsum light gray, venter cream colored. ***Wings*** – Forewing dorsal ground color a mixture of gray-brown, reddish-brown and beige-colored scales; veins lined with gray, especially distally; anal fold and cubitus reddish brown; orbicular spot diffuse reddish brown; reniform spot small, reddish brown; medial M line thin, reddish brown, a wide, vaguely-defined beige band beyond it; postmedial PM line thin, reddish brown, poorly defined; adterminal AD line reddish brown, fringe gray brown. Ventral surfaces of both wings gray brown. Dorsal hindwing dirty gray brown, lighter near base (Figs 7, 8). (WL 10.70–13.3 mm). ***Male genitalia*** (Figs 11–13) – T8 quadrate, posterior margin narrowly sclerotized; St8 wide, short, anterior margin simple, posterior margin bearing a pair of small, widely separated processes, a second, longer pair of processes arise between arms of fork (Fig. 11). Uncus lobule-like, short and pubescent; socci thin, short and slightly curved. Valva broad and membranous, with saccular margin serrate and inner surface with spine-like process, near appex (Fig. 12). Phallus thin basally, wider medially with a pair of short, serrate projections on each margin, distal part with a pair of opposite marginal non-serrate projections, longer than anterior ones. Vesica short, unsclerotized (Fig. 13). **Female** (Figs 9, 10, 14). Antenna filiform, shaft cream with a mix of reddish- and gray-brown scales; body color and wing pattern similar to male, wings longer and darker (Figs 9, 10). (WL 13.7–14.7 mm). ***Female genitalia*** (Fig. 14) – St8 forming a heavily sclerotized capsule; anterior apophyses thin and acute; posterior apophyses thin, CB small and rounded, signum absent; DB short; ostium recessed in St8. Ovipositor lobes triangulate and setose.

#### Natural history

(Figs 74, 79, 80, 81). 506 rearing records: ACG locations: Sector Del Oro (n=84), Orosi (n=4), Pitilla (n=68), Rincon Rain Forest (n=132), San Cristobal (n=217); all mid-elevation rain forest and does not occur in ACG dry forest either as caterpillars or free-flying adults.

Food plants: exclusively Arecaceae: *Asterogyne martiana* (H. Wendl.) H. Wendl. ex Hemsel (n=9), *Astrocaryum alatum* F.H. Loomis (n=5), *Calyptrogyne trichostachys* Burret (n=3), *Chamaedorea pinnatifrons* (Jacq.) Oerst. (n=14), *Chamaedorea tepejilote* Liebm. (n=298), *Chamaedorea warscewiczii* H. Wendl. (n=1) *Cryosophila warscewiczii* (H. Wendl.) Bartlett (n=49), *Geonoma congesta* H. Wendl. ex Spruce (n=1), *Geonoma cuneata* H. Wendl. ex Spruce (n=3), *Geonoma ferruginea* H. Wendl. ex Spruce (n=42), *Geonoma interrupta* (Ruiz & Pav.) Marz. (n=5), *Iriartea deltoidea* Ruiz & Pav. (n=41), *Prestoea decurrens* (H. Wendl. ex Burret) H.E. Moore (n=21), *Welfia regia* H. Wendl. (n=14).

Eggs laid in small batches of 5–40, and caterpillars may remain together through the penultimate instar, but generally forage separately in the last instar. Cocoons are solitary, generally in a fold of the palm leaf or two pinnae one on top of the other, lightly silked together. The relatively conspicuous caterpillars remain on the leaf when disturbed rather than drop to the ground, implying that they may be aposematic or mimetic even though they are commonly difficult to encounter among overlapping leaf parts, and often on the underside of the leaf.

Altitude (meters): 340, 405, 420, 645, 680.

#### Parasitoids.

27 records from 506 wild-caught caterpillars over 24 years of rain forest search. **Braconidae:** Macrocentrinae: *Austrozele* Janzen03 (n=6) DHJPAR0029342, DHJPAR0029346, DHJPAR0029344, DHJPAR0029347, DHJPAR0029378, DHJPAR0029377; shared only with *Dunama mexicana*DHJ01. Microgastrinae: *Diolcogaster* Choi71 (n=1) DHJPAR0004716; unique to this species of caterpillar. **Tachinidae:** *Calolydella* Wood01DHJ06 (n=13) DHJPAR0017779, DHJPAR0017778, DHJPAR0017777, DHJPAR0017781, DHJPAR0017780, DHJPAR0007021, etc., which it shares with 4 species of *Dioptis* Hübner, *Dottia* Schaus and *Tithraustes* Druce (26 total rearings of this fly), which are similar-sized notodontids that eat the same species of palms in the same forest; *Lespesia* Wood33DHJ06 (n=3) DHJPAR0037477, DHJPAR0037483, DHJPAR0037482, which it shares with six other species of similar-sized notodontids*Dunama* (n=4), *Dottia* (n=1), and *Heorta* Walker (n=1)feeding on the same palms in the same rain forest habitat;four nematodes and two fungi.

#### Hyperparasitoids.

One puparium of *Calolydella* Wood01DHJ06 was hyperparasitized by *Taeniogonalos woodorum* Smith (DHJPAR0010604), Trigonalidae (Smith et al. 2012).

#### Distribution.

Adults of *Dunama jessiehillae* have been collected on the east slope of Cordillera Volcanica de Guanacaste and Tilaran, and in the Sarapiqui lowlands, from 40 to 1500 m elevation (Fig. 85), but larvae have only been encountered at mid-elevations on the same slopes.

#### Remarks.

This species feeds exclusively on Arecaceae. Several barcode haplotypes are present in populations from La Selva, Heredia west to the Pacific coast, but they are all very similar (Fig. 86). The most common haplotype is shared with *Dunama angulinea*, which ocupies the Caribbean coastal area and differs only in the structure of its sternum. We elected on that basis to describe it as a new species, following both the advice of a reviewer and our own analysis. If later studies support the doubtful hypothesis that this is merely geographic variation in a widely distributed species, then *Dunama jessiehillae* would be synonymized with *Dunama angulinea*.

### Dunama jessiebarronae

Chacón sp. n.

urn:lsid:zoobank.org:act:F073A2EC-C71B-49C5-B601-350005110680

http://species-id.net/wiki/Dunama_jessiebarronae

Figs 15–22 , 65–70

#### Type material.

**Holotype** male: 04-SRNP-4063 (Dissected, COI Barcoded) Costa Rica. Prov. Alajuela. Sector San Cristobal, Puente Palma 10.9163, -85.37869, 460m. 7 September 2004. Elda Araya (INBio). **Paratypes**:2♂ 1♀. Female:04-SRNP-4060 (Dissected, COI Barcoded), Costa Rica, Prov. Alajuela, Sector San Cristobal, Puente Palma 10.9163, -85.37869, 460 m, 5 September 2004, Elda Araya (INBio). Female: 00-SRNP-1959, Costa Rica, Prov. Alajuela, Sector San Cristobal, Rio Blanco Abajo 10.90037, -85.37254, 500 m, 26 May 2000, Osvaldo Espinoza. Male: 00-SRNP-1935 (COI Barcoded), Costa Rica, Prov. Alajuela, Sector San Cristobal, Rio Blanco Abajo 10.90037, -85.37254, 500 m, 26 May 2000, Osvaldo Espinoza.

#### Other material examined.

Barcoded: 25 specimens that divided into 6 apparent haplotypes with differences from the most common haplotype (16 specimens) of 0.3% or less (Fig. 86). Barcoded specimens were from Alajuela and Limon Provinces. Many hundreds more of this species were reared and barcoded, but there is no cause to list them here. Museum specimens: (67 specimens) 22♂ 26♀ Alajuela, 13♂ Heredia, 6♂ Limon. Dissections: 4♂ 5♀ Alajuela, 2♂ Heredia, 5♂ Limon. INBio, USNM, JBS. **Janzen & Hallwachs vouchers of reared specimens**:Female: 00-SRNP-1940, Costa Rica, Prov. Alajuela, Sector San Cristobal, Rio Blanco Abajo 10.90037, -85.37254, 500 m, 26 May 2000, Osvaldo Espinoza. Male: 00-SRNP-1942, Costa Rica, Prov. Alajuela, Sector San Cristobal, Rio Blanco Abajo 10.90037, -85.37254, 500 m, 26 May 2000, Osvaldo Espinoza. Female: 00-SRNP-1943, Costa Rica, Prov. Alajuela, Sector San Cristobal, Rio Blanco Abajo 10.90037, -85.37254, 500 m, 26 May 2000, Osvaldo Espinoza. Female: 00-SRNP-1959, Costa Rica, Prov. Alajuela, Sector San Cristobal, Rio Blanco Abajo 10.90037, -85.37254, 500 m, 26 May 2000, Osvaldo Espinoza. Male:00-SRNP-1967, Costa Rica, Prov. Alajuela, Sector San Cristobal, Rio Blanco Abajo 10.90037, -85.37254, 500 m, 23 May 2000, Osvaldo Espinoza. Female: 01-SRNP-4001, Costa Rica, Prov. Alajuela, Sector Rincon Rain Forest, Camino Rio Francia 10.90425, -85.28651, 410 m, 12 January 2001, Jose Perez. Female: 01-SRNP-4003, Costa Rica, Prov. Alajuela, Sector Rincon Rain Forest, Camino Rio Francia 10.90425, -85.28651, 410 m, 12 January 2001, Jose Perez. Female: 01-SRNP-4006, Costa Rica, Prov. Alajuela, Sector Rincon Rain Forest, Camino Rio Francia 10.90425, -85.28651, 410 m, 12 January 2001, Jose Perez. Male: 01-SRNP-4008, Costa Rica, Prov. Alajuela, Sector Rincon Rain Forest, Camino Rio Francia 10.90425, -85.28651, 410 m, 11 January 2001, Jose Perez. Female: 01-SRNP-4001,Costa Rica, Prov. Alajuela, Sector Rincon Rain Forest, Camino Rio Francia 10.90425, -85.28651, 410 m, 12 January 2001, Jose Perez. Male: 01-SRNP-4177,Costa Rica, Prov. Alajuela, Sector Rincon Rain Forest, Quebrada Escondida 10.89928, -85.27486, 420 m, 5 February 2001, Jose Perez. Female: 01-SRNP-23045,Costa Rica, Prov. Alajuela, Sector Rincon Rain Forest, Montaña Figueres 10.88367, -85.29081, 460 m, 1 November 2001, Jose Perez. Male: 04-SRNP-4056, Costa Rica, Prov. Alajuela, Sector San Cristobal, Puente Palma 10.9163, -85.37869, 460 m, 5 September 2004, Elda Araya. Female: 07-SRNP-1085, Costa Rica, Prov. Alajuela, Sector San Cristobal, Puente Palma 10.9163, -85.37869, 460 m, 20 March 2007, Osvaldo Espinoza. **INBio specimens**: Male: INBIOCRI000702122 (Dissected), Costa Rica, Prov. Heredia,P. N. Braulio Carrillo, Est. Magsasay 10.401255, -84.049314, 200 m, January 1991, M. Barrelier. Male: INB0003319795, Costa Rica, Prov. Limon, Valle La Estrella, R. B.Hitoy Cerere, Est. Hitoy Cerere 9.671035, -83.026156, 100 m, November 2001, L. Chavarria. Male: INB0003558871 (Dissected), Costa Rica, Prov. Heredia, Fca. La Selva, Puerto Viejo de Sarapiqui 10.431958, -84.0091, 55 m, 4 August 1981, D.H. Janzen & W. Hallwachs. Male: INB0004268509, Costa Rica, Prov. Alajuela, San Ramon, Est. Biol. Villa Blanca 10.201361, -84.485101, 1115 m, September 2009, R. Rojas (Reared). Male: INB0004268510, Costa Rica, Prov. Alajuela, San Ramon, Est. Biol. Villa Blanca 10.201361, -84.485101, 1115 m, September 2009, R. Rojas (Reared). Male: INB0004251494 (Dissected), Costa Rica, Prov. Alajuela, San Ramon, Est. Biol. Villa Blanca 10.201361, -84.485101, 1115 m, August 2009, R. Rojas (Reared). Male: INB0004251489, Costa Rica, Prov. Alajuela, San Ramon, Est. Biol. Villa Blanca 10.201361, -84.485101, 1115 m, July 2009, R. Rojas (Reared). Male: INB0004251485, Costa Rica, Prov. Alajuela, San Ramon, Est. Biol. Villa Blanca 10.201361, -84.485101, 1115 m, July 2009, R. Rojas (Reared). Male: INB0004251493, Costa Rica, Prov. Alajuela, San Ramon. Est. Biol. Villa Blanca 10.201361, -84.485101, 1115 m, August 2009, R. Rojas (Reared). Male: INB0004251492, Costa Rica, Prov. Alajuela, San Ramon, Est. Biol. Villa Blanca 10.201361, -84.485101, 1115 m, August 2009, R. Rojas (Reared). Male: INB0004251491, Costa Rica, Prov. Alajuela, San Ramon, Est. Biol. Villa Blanca 10.201361, -84.485101, 1115 m, August 2009, R. Rojas (Reared). Male: INB0004251496, Costa Rica, Prov. Alajuela, San Ramon, Est. Biol. Villa Blanca 10.201361, -84.485101, 1115 m, August 2009, R. Rojas (Reared). Male: INB0004251495, Costa Rica, Prov. Alajuela, San Ramon, Est. Biol. Villa Blanca 10.201361, -84.485101, 1115 m, August 2009, R. Rojas (Reared). Male: INB0004251494 (Dissected), Costa Rica, Prov. Alajuela, San Ramon, Est. Biol. Villa Blanca 10.201361, -84.485101, 1115 m, 12 July 2010, M. Gutierrez, R. Rojas (Reared). Male:INB0004301720 (Dissected), Costa Rica, Prov. Alajuela, San Ramon, Est. Biol. Villa Blanca 10.201361, -84.485101, 1115 m, August 2009, R. Rojas (Reared). Female: INBIOCRI002112258, Costa Rica, Prov. Alajuela, San Ramon 10.224969, -84.587984, 800 m, September 1994, G. Carballo. Female: INB0004301719 (Dissected), Costa Rica, Prov. Alajuela, San Ramon, Est. Biol. Villa Blanca 10.201361, -84.485101, 1115 m, 12 July 2010, M. Gutierrez, R. Rojas (Reared). Female: INB0004268508, Costa Rica, Prov. Alajuela, San Ramon, Est. Biol. Villa Blanca 10.201361, -84.485101, 1115 m, September 2009, R. Rojas (Reared). Female: INB0004268511, Costa Rica, Prov. Alajuela, San Ramon, Est. Biol. Villa Blanca 10.201361, -84.485101, 1115 m, September 2009, R. Rojas (Reared). Female: INB0004251484 (Dissected), Costa Rica, Prov. Alajuela, San Ramon, Est. Biol. Villa Blanca 10.201361, -84.485101, 1115 m, July 2009, R. Rojas (Reared). Female: INB0004251497, Costa Rica, Prov. Alajuela, San Ramon, Est. Biol. Villa Blanca 10.201361, -84.485101, 1115 m, August 2009, R. Rojas (Reared). Female: INB0004251486, Costa Rica, Prov. Alajuela, San Ramon, Est. Biol. Villa Blanca 10.201361, -84.485101, 1115 m, July 2009, R. Rojas (Reared). Female: INB0004251488, Costa Rica, Prov. Alajuela, San Ramon, Est. Biol. Villa Blanca 10.201361, -84.485101, 1115 m, July 2009, R. Rojas (Reared). Female: INB0004251487, Costa Rica, Prov. Alajuela, San Ramon, Est. Biol. Villa Blanca 10.201361, -84.485101, 1115 m, July 2009, R. Rojas (Reared). Female: INB0004301721 (Dissected), Costa Rica, Prov. Alajuela, San Ramon, Est. Biol. Villa Blanca 10.201361, -84.485101, 1115 m, 14 June 2010, M. Gutierrez (Reared). Female: INB0004301704, Costa Rica, Prov. Alajuela, San Ramon, Est. Biol. Villa Blanca 10.201361, -84.485101, 1115 m, 12 July 2010, M. Gutierrez, R. Rojas (Reared). Female: INB0004301705, Costa Rica, Prov. Alajuela, San Ramon, Est. Biol. Villa Blanca 10.201361, -84.485101, 1115 m, 12 July 2010, M. Gutierrez, R. Rojas (Reared). Female: INB0004301706, Costa Rica, Prov. Alajuela, San Ramon, Est. Biol. Villa Blanca 10.201361, -84.485101, 1115 m, 12 July 2010, M. Gutierrez, R. Rojas (Reared). Female: INB0004301707, Costa Rica, Prov. Alajuela, San Ramon, Est. Biol. Villa Blanca 10.201361, -84.485101, 1115 m, 12 July 2010, M. Gutierrez, R. Rojas (Reared). Female: INB0004301708, Costa Rica, Prov. Alajuela, San Ramon, Est. Biol. Villa Blanca 10.201361, -84.485101, 1115 m, 12 July 2010, M. Gutierrez, R. Rojas (Reared). Female: INB0004301709, Costa Rica, Prov. Alajuela, San Ramon, Est. Biol. Villa Blanca 10.201361, -84.485101, 1115 m, 12 July 2010, M. Gutierrez, R. Rojas (Reared). Female: INB0004301710, Costa Rica, Prov. Alajuela, San Ramon, Est. Biol. Villa Blanca 10.201361, -84.485101, 1115 m, 12 July 2010, M. Gutierrez, R. Rojas (Reared). Female: INB0004301711, Costa Rica, Prov. Alajuela, San Ramon, Est. Biol. Villa Blanca 10.201361, -84.485101, 1115 m, 12 July 2010, M. Gutierrez, R. Rojas (Reared). Female: INB0004301712, Costa Rica, Prov. Alajuela, San Ramon, Est. Biol. Villa Blanca 10.201361, -84.485101, 1115 m, 12 July 2010, M. Gutierrez, R. Rojas (Reared). Female: INB0004301713, Costa Rica, Prov. Alajuela, San Ramon, Est. Biol. Villa Blanca 10.201361, -84.485101, 1115 m, 12 July 2010, M. Gutierrez, R. Rojas (Reared). Female: INB0004301714, Costa Rica, Prov. Alajuela, San Ramon, Est. Biol. Villa Blanca 10.201361, -84.485101, 1115 m, 12 July 2010, M. Gutierrez, R. Rojas (Reared). Female: INB0004301715, Costa Rica, Prov. Alajuela, San Ramon, Est. Biol. Villa Blanca. 10.201361, -84.485101, 1115 m, 12 July 2010. M. Gutierrez, R. Rojas (Reared). Female: INB0004301716, Costa Rica, Prov. Alajuela, San Ramon, Est. Biol. Villa Blanca 10.201361, -84.485101, 1115 m, 12 July 2010, M. Gutierrez, R. Rojas (Reared). Female: INB0004301717, Costa Rica, Prov. Alajuela, San Ramon, Est. Biol. Villa Blanca 10.201361, -84.485101, 1115 m, 12 July 2010, M. Gutierrez, R. Rojas (Reared). Female: INB0004301718, Costa Rica, Prov. Alajuela, San Ramon, Est. Biol. Villa Blanca 10.201361, -84.485101, 1115 m, 12 July 2010, M. Gutierrez, R. Rojas (Reared).

#### Etymology.

This species is named in honor of Ms. Jessie Barron, great-great-grandmother of Jessie Hill of Philadelphia and Hawaii, and in emphatic recognition of Jessie Hill’s contribution to saving and inventorying the conserved ACG rain forest in which *Dunama jessiebarronae* breeds.

#### Diagnosis.

St8 wide, short, anterior margin simple, posterior margin bearing a pair of long, widely separated processes with serrate margins. Phallus thin at the base, wider and sclerotized in the distal part, with a pair of opposite basal projections at the base of the sclerotized portion and a small subterminal projection distally on the left side and a small finger-like terminal projection.

#### Description.

**Figures 15–22.**
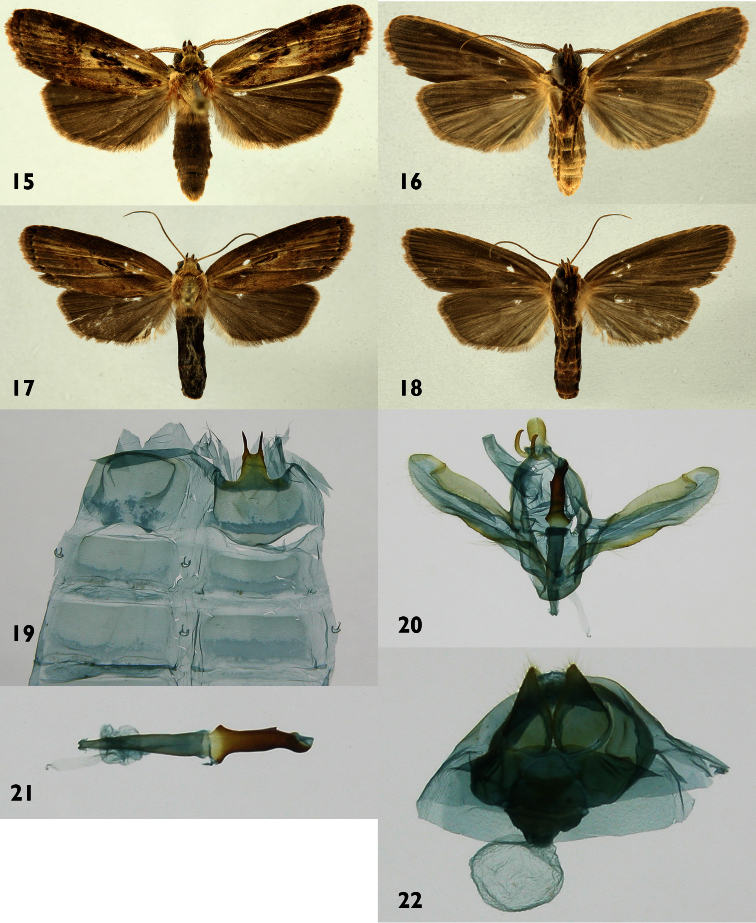
*Dunama jessiebarronae* **15, 16** Male dorsal and ventral 04-SRNP-4063 **17, 18** Female dorsal and ventral 04-SRNP-4060 **19** Male St8 **20** Male genitalia 04-SRNP-4063 **21** Phallus **22** Female genitalia04-SRNP-4060.

**Male** (Figs 15, 16, 19–21). ***Head*** – Antenna pectinate in basal 4/5, rami moderately long, black, distal fifth simple, shaft cream with a few reddish-brown scales, scape with scale tuft reddish brown and creamy; haustellum well developed, frons with a mix of creamy and reddish-brown scales; labial palpus upcurved, blackish brown with a few scattered cream-colored scales; ocelli absent; vertex cream, reddish brown laterally; patagium reddish brown near midline, blackish brown laterally, margins cream colored. ***Thorax and abdomen*** – Tegula cream colored at the base, a mix of cream and reddish-brown scales distally, mesoscutum reddish brown anteriorly, cream and reddish brown posteriorly, mesoscutellum mostly creamy white; thoracic pleuron cream colored to reddish brown; legs mostly reddish brown on outer surfaces, cream colored on inner ones. Abdominal dorsum light gray, venter cream colored. ***Wings*** – Forewing dorsal ground color cream at base, 2/3 black brown; a prominent black-brown, slightly oblique bar between base of reniform spot and base of inner margin of forewing in both sexes; veins lined with gray, especially distally; anal fold and cubitus blackish brown; orbicular spot diffuse, blackish brown; reniform spot small, blackish brown; M-line thin, blackish brown, a wide, vaguely-defined beige band beyond it; AD-line with blackish-brown spots, fringe gray brown. Ventral surfaces of both wings gray brown. Forewing costal margin cream ventrally. Hindwing dorsal dirty gray brown, lighter near base (Figs 15, 16). (WL 13.5–13.7 mm). ***Male genitalia*** (Figs 19–21) – T8 quadrate, posterior margin narrrowly sclerotized; St8 wide, short, anterior end with simple margin, posterior margin bearing a pair of long, widely-separated processes with serrate margins (Fig. 19). Uncus elongated lobule-like and lightly pubescent, socci thin and curved. Valva broad and membranous, with slightly undulating serrate saccular margin, inner surface with a small spine-like process near apex (Fig. 20). Phallus thin at base, widening and sclerotized distally, with a pair of opposite basal projections at base of sclerotized portion, which has a small subterminal projection on left side and a small finger-like terminal projection (Fig. 21). Vesica small. **Female** (Figs 17, 18, 22). Antenna filiform, shaft cream; body color and wing pattern similar to male but wings longer and darker (Figs 17, 18). (WL 16.6–17.2 mm). ***Female genitalia*** (Fig. 22) – Segment 8 forming a heavily sclerotized capsule; anterior apophyses acute; posterior apophyses tiny, CB small and rounded, signum absent; DB short; ostium recessed in St8. Ovipositor lobes acute and setose.

#### Natural history

(Figs 65–70). 489 rearing records, all from intermediate elevation rain forest in ACG.

Food plants: Heliconiaceae:*Heliconia irrasa* R.R Sm. (n=169).* Heliconia latispatha* Benth. (n=126), *Heliconia pogonantha* Cufod. (n=9); Musaceae; *Musa acuminata* Colla (introduced) (n=185). All other *Dunama* spp. food plant records are from Arecaceae, and there are no *Dunama jessiebarronae* reared from Arecaceae.

#### Parasitoids.

**Tachinidae**: out of 489 rearing records of wild-caught caterpillars, 10 records generated *Lespesia* Wood33DHJ06, DHJPAR0015889, DHJPAR0015890, DHJPAR0015880, DHJPAR0015878, DHJPAR0015903, DHJPAR0008353, DHJPAR0015879, DHJPAR0015875, DHJPAR0015876, DHJPAR0015882.This fly parasitizes only Notodontidae feeding on Heliconiaceae and Musaceae, and also parasitizes three other species of *Dunama* feeding on Arecaceae, as well as *Dottia* and *Heorta* feeding on the same family. A single record (DHJPAR0016614**)** of *Lespesia* Wood03bDHJ05 parasitizing *Dunama jessibaronae* is not surprising, given that this fly parasitzes five caterpillar families feeding on a large variety of plant species and families, and as such is quite “generalist.”

#### Distribution.

*Dunama jessiebarronae*has been collected on the east slope of Cordillera Volcanica de Guanacaste, Cordillera Volcanica Central, Cordillera de Talamanca, llanuras de Sarapiqui, and the lowlands of the Caribbean, from 50 to 1115 m elevation (Fig. 85).

#### Remarks.

Adults of *Dunama jessiebarronae* have almost the exact maculation pattern of *Dunama tuna* (Schaus), but the latter moth is larger.In addition, there are two lateral prongs at the base of the posterior projection of the sternum. Todd (1976) made *Dunama sagittula* (Dognin, 1914), a Colombian species, a synonym of *Dunama tuna*, whose type locality is also in Colombia. Although he did not dissect the type of *Dunama tuna*, we did, and it agrees with that of *Dunama sagittula*. Todd also reported a male specimen from Porto Bello, Panama and from Sixaola River, Costa Rica. These two Central American specimens are smaller than are those from Colombia, and they also have lateral prongs on the sternum, which are absent in all Costa Rican specimens of *Dunama jessiebarronae* we have examined, including 2 males from Hitoy Cerere, Limon Province, which is located not far from Sixaola River. Because we were unable to find any recent specimens with lateral prongs on the tergum, and because barcoding has shown few species in common between Costa Rica and northern South America (unpublished), we have chosen to treat the Costa Rican populations as a new species, *Dunama jessiebarronae*. The specimens from Sixaola River and Panama cited by Todd are believed to be *Dunama jessiebarronae*. Changes in the form of the sternum along the Atlantic Coast are also seen in the closely related species *Dunama jessiehillae* and *Dunama angulinea*, albiet it is the reverse change. Future examination and barcoding of specimens collected in Panama and northern Colombia should resolve this issue.

### Dunama janewaldronae

Chacón sp. n.

urn:lsid:zoobank.org:act:6A297DF2-4FB7-4D49-AD12-6759563E8240

http://species-id.net/wiki/Dunama_janewaldronae

Figs 23–30 , 75–78

#### Type material.

**Holotype** male: 08-SRNP-430 (Dissected, COI Barcoded), Costa Rica, Alajuela, Sector San Cristobal, Suampo Uncaria 10.93597, -85.37135, 506 m, 16 February 2008, Gloria Sihezar. **Paratypes** (all reared from wild-caught caterpillars):(2♂ 2♀).Male:08-SRNP-382(COI Barcoded),Costa Rica, Prov. Alajuela, Sector San Cristobal, Suampo Uncaria 10.93597, -85.37135, 506 m, 16 February 2008, Gloria Sihezar. Male: 08-SRNP-433 (Dissected, COI Barcoded),Costa Rica, Prov. Alajuela, Sector San Cristobal, Suampo Uncaria 10.93597, -85.37135, 506 m, 16 February 2008, Gloria Sihezar. Female:08-SRNP-407(COI Barcoded),Costa Rica, Prov. Alajuela, Sector San Cristobal, Suampo Uncaria 10.93597, -85.37135, 506 m, 16 February 2008, Gloria Sihezar. Female: 00-SRNP-21518 (Dissected, COI Barcoded),Costa Rica, Prov. Alajuela, Sector San Cristobal, Rio Blanco Abajo 10.90037, -85.37254, 500 m, 5 November 2000, Freddy Quesada.

#### Other material examined.

Barcoded: 78 specimens, which divided into four haplotypes with differences from the most common haplotype (44 specimens) of 0.25% or less, except for the specimens from Limon that were idéntical, or about 0.6% different. Barcoded specimens were from Alajuela and Limon Provinces (Fig. 86). Museum specimens: (18): 2♂ 1♀ Guanacaste, 4♂ 6♀ Alajuela, 1♂ Heredia, 4♂ Limon. Dissections: 2♂ 1♀ Guanacaste, 1♂ 1♀ Alajuela, 1♂ Heredia, 1♂ Limon.

**Janzen & Hallwachs voucher specimens**: Male: 08-SRNP-390 (COI Barcoded), Costa Rica, Prov. Alajuela, Sector San Cristobal, Suampo Uncaria 10.93597, -85.37135, 506 m, 16 February 2008, Gloria Sihezar. Male: 00-SRNP-21521, Costa Rica, Prov. Alajuela, Sector San Cristobal, Rio Blanco Abajo 10.90037, -85.37254, 500 m, 23 October 2000, Freddy Quesada. Female: 08-SRNP-399 (Dissected, COI Barcoded), Costa Rica, Prov. Alajuela, Sector San Cristobal, Suampo Uncaria 10.93597, -85.37135, 506 m, 15 February 2008, Gloria Sihezar. Female: 08-SRNP-419 (COI Barcoded), Costa Rica, Prov. Alajuela, Sector San Cristobal, Suampo Uncaria 10.93597, -85.37135, 506 m, 15 February 2008, Gloria Sihezar. Female: 08-SRNP-423 (COI Barcoded), Costa Rica, Prov. Alajuela, Sector San Cristobal, Suampo Uncaria 10.93597, -85.37135, 506 m, 16 February 2008, Gloria Sihezar.

**INBio specimens**: Male: INBIOCRI001288520 (Dissected, COI Barcoded), Costa Rica, Prov. Guanacaste,P. N. Guanacaste, 9 Km S Santa Cecilia, Est. Pitilla 10.992609, -85.429477, 700 m, 30 January 1993, P. Rios. Female: INBIOCRI002583021, Costa Rica, Prov. Alajuela, Santa Cecilia, 8 Km S. Estacion Pitilla 10.990808 -85427641, 680 m, February 1988, A. Chacon & M. Espinoza.

#### Etymology.

This species is named in honor of Ms. Jane Waldron, great-grandmother of Jessie Hill of Philadelphia and Hawaii, and in emphatic recognition of Jessie Hill’s contribution to saving and inventorying the conserved ACG rain forest in which *Dunama janewaldronae* breeds.

#### Diagnosis.

St8 wide, short, anterior margin simple, posterior margin bearing a pair of small, widely separated processes with serrate margins, a second, long pair of processes arising between arms of first pair. Phallus thin in the base, wider and sclerotized in distal part, ventral margin of distal part with six teeth and dorsal margin with two small teeth.

#### Description.

**Figures 23–30.**
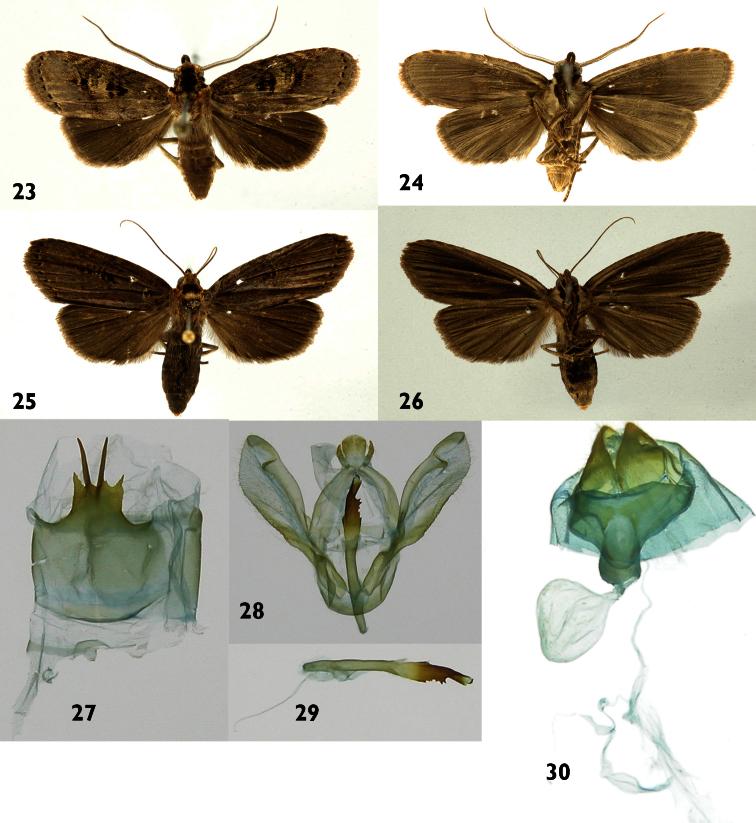
*Dunama janewaldronae* **23, 24** Male dorsal and ventral 08-SRNP-430 **25, 26** Female dorsal and ventral 00-SRNP-21518 **27** Male St8 **28** Male genitalia 08-SRNP-430 **29** Phallus **30** Female genitalia 00-SRNP-21518.

**Male** (Figs 23, 24, 27–29). ***Head*** – Antenna pectinate in basal 4/5, rami moderately long reddish brown, distal fifth simple, shaft with a mix of reddish-brown and gray brown scales, scape with scale tuft blackish brown at base and cream to tip; frons with a mix of cream and blackish-brown scales; labial palpus upcurved blackish brown with a few scattered cream-colored scales; ocelli absent; vertex blackish brown, cream colored laterally; patagium blackish brown near midline, blackish brown laterally, margins cream colored. ***Thorax and abdomen*** – Tegula cream colored at base, a mix of cream and blackish-brown scales distally; mesoscutum blackish brown anteriorly, cream and reddish brown posteriorly; mesoscutellum mostly creamy white; thoracic pleuron cream colored to blackish brown; legs mostly blackish brown on outer surfaces, cream colored on inner surfaces. Abdominal dorsum light gray, venter cream colored. ***Wings*** – Dorsal ground color a mixture of gray-brown and beige scales; veins lined with gray, especially distally; anal fold and cubitus blackish brown; orbicular spot diffuse blackish brown; reniform spot small, blackish brown; M-line thin, blackish brown, a wide, vaguely-defined beige band beyond it; PM-line thin, blackish brown, poorly defined; AD-line with blackish-brown spots, fringe gray brown. Ventral surfaces of both wings gray brown. Dorsal hindwing dirty gray brown, lighter near base (Figs 23, 24). (WL 12.5–13.4 mm). ***Male genitalia*** (Figs 27–29) – St8 wide, short, anterior margin simple, posterior margin bearing a pair of small, widely separated processes with serrate margins; a second, long pair of processes arising between arms of first pair (Fig. 27). Uncus lobule-like and lightly pubescent, socci thin and lightly curved. Valva broad and membranous, with serrate saccular margin, inner surface with a hook-like process near apex (Fig. 28). Phallus thin at base, wider and sclerotized distally, ventral margin of distal part with six teeth and dorsal margin with two small teeth. Vesica tiny, bearing a minute cornutus (Fig. 29). **Female** (Figs 25, 26, 30). Antenna filiform, shaft gray brown; body color and wing pattern similar to male but wings longer and darker (Figs 25, 26). (WL 16.5–16.8 mm). ***Female genitalia*** (Fig. 30) – Segment 8 forming a heavily sclerotized capsule; anterior apophyses acute; posterior apophyses tiny, CB small and rounded, signum absent; DB short; ostium recessed in St8. Ovipositor lobes triangulate and setose.

#### Natural history 

(Figs 75, 76, 77, 78). 201 records reared from Sector Pitilla (n=13), Rincon Rain Forest (n=57), and San Cristobal (n=131), all rain forest sites.

Food plants: Arecaceae: *Chamaedorea dammeriana* Burret (n=42), *Geonoma congesta* (n=22), *Geonoma cuneata* (n=114), *Prestoea decurrens* (n=13), *Welfia regia* (n=10).

#### Parasitoids

: **Braconidae**: Macrocentrinae, *Austrozele* Janzen03 (n=5), shared with *Dunama jessiebarronae*. **Tachinidae**: *Lespesia* Wood33DHJ06 (n=7), shared with *Dunama jessiebarronae*, and *Jurinella* Wood06 (n=1). The latter species parasitizes only Notodontidae and Hesperiidae feeding on rain forest Arecaceae.

#### Distribution.

*Dunama janewaldronae* has been reared from intermediate elevations of the eastern side of the Cordillera Volcanica de Guanacaste from 400 to 680 m elevation (Fig. 85).

#### Remarks.

This species shows identical genitalia and very similar barcodes throughout Costa Rica (Fig. 86). Specimens from the Caribbean side of Costa Rica have the most divergent barcodes, but are still within the range of variation seen for most species. Nearest neighbor analyses pair *Dunama janewaldronae* with *Dunama angulinea* and they differ mostly in being slightly different in size. They share several species of understory Arecaceae as caterpillar food plants.

### Dunama jessiebancroftae

Chacón sp. n.

urn:lsid:zoobank.org:act:954407D9-FA83-44B5-9BEA-EEE1612AA585

http://species-id.net/wiki/Dunama_jessiebancroftae

Figs 31–38 , 82–84

#### Type material.

**Holotype** male: 09-SRNP-56330 (Dissected, COI Barcoded), Costa Rica, Prov. Guanacaste, Sector Pailas, Gemelos 10.76928, -85.34662, 1276 m, 18 June 2009, Daniel M. Acuna (INBio). **Paratypes**:2♂ 2♀. Male: 06-SRNP-36778 (COI Barcoded), Costa Rica, Prov. Guanacaste, Sector Cacao, Sendero Abajo 10.92547, -85.47158, 1020 m, 12 December 2006, Harry Ramirez. Male: 08-SRNP-57204, Costa Rica, Prov. Guanacaste, Sector Mundo Nuevo, Vado Chamaedorea 10.77638, -85.40024, 570 m, 16 August 2008, Mariano Pereira. Female: 06-SRNP-47624 (COI Barcoded), Costa Rica, Prov. Guanacaste, Sector Cacao, Puente Gongora 10.88489, -85.47203, 540 m, 10 September 2006, Dunia Garcia. Female: 06-SRNP-36773 (Dissected, COI Barcoded), Costa Rica, Prov. Guanacaste, Sector Cacao, Sendero Abajo 10.92547, -85.47158, 1020 m, 12 October 2006, Harry Ramirez. Female: 08-SRNP-57269 (COI Barcoded), Costa Rica, Prov. Guanacaste, Sector Mundo Nuevo, Vado Chamaedorea 10.77638, -85.40024, 570 m, 15 August 2008, Jose Cortez.

#### Other material examined.

Barcoded: 75 specimens from Guanacaste and Puntarenas Provinces that divide into 3 principal haplotypes (excluding 2 partial sequences), which differed from each other by less than 0.25%. One haplotype predominated (66 specimens). Museum specimens: 6 specimens: 3♂ 2♀ Guanacaste, 1♂ Puntarenas. Dissections: 2♂ 1♀ Guanacaste, 1♂ Puntarenas. **Janzen & Hallwachs voucher specimens**: Male: 08-SRNP-57752 (Dissected, COI Barcoded), Costa Rica, Prov. Guanacaste, Sector Mundo Nuevo, Vado Chepon 10.77816, -85.41629, 440 m, 10 October 2008, Jose Cortez. Male: 06-SRNP-47625 (COI Barcoded), Costa Rica, Prov. Guanacaste, Sector Cacao, Puente Gongora 10.88489, -85.47203, 540 m, 10 September 2006, Dunia Garcia. Female: 08-SRNP-57739 (Dissected, COI Barcoded), Costa Rica, Prov. Guanacaste, Sector Mundo Nuevo, Vado Chepon 10.77816, -85.41629, 440 m, 8 October 2008, Jose Cortez. Female: 09-SRNP-56324 (COI Barcoded), Costa Rica, Prov. Guanacaste, Sector Pailas, Gemelos 10.76928, -85.34662, 1276 m, 17 July 2009, Daniel M. Acuña. **INBio specimens**: Male: INB0003435267 (Dissected, COI Barcoded), Costa Rica, Prov. Guanacaste, Z.P. Nosara, Cerro Romo 10.002648, -85.404627, 885 m, 10–15 February 2002, H. Mendez. Male: INBIOCRI0020454417 (Dissected, COI Barcoded), Costa Rica, Prov. Puntarenas, R.B. Carara, Quebrada Bonita 9.774233, -84.608124, 50 m, October 1994, J.C. Saborio.

#### Etymology.

This species is named in honor of Ms. Jessie Bancroft, grandmother of Jessie Hill of Philadelphia and Hawaii, and in emphatic recognition of Jessie Hill’s contribution to saving and inventorying the conserved ACG rain forest in which *Dunama jessiebancroftae* breeds.

#### Diagnosis.

St8 wide and short, anterior margin simple, posterior margin bearing a simple acute and triangulate process. Phallus narrow at base, expanding medially, heavily sclerotized at distal third, with dorsal margin serrate, narrowing to tip.

#### Description.

**Figures 31–38.**
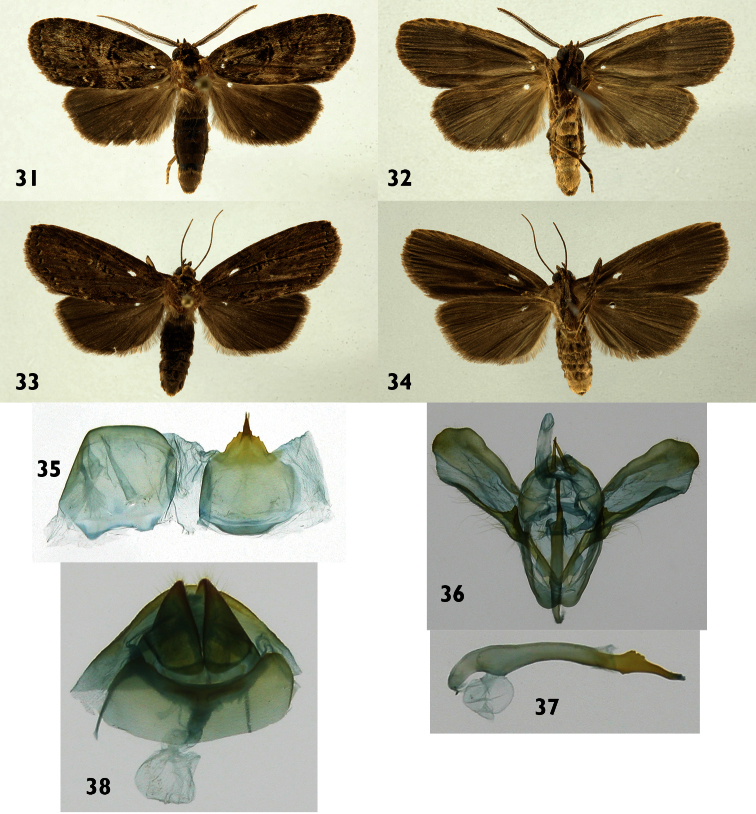
*Dunama jessiebancroftae* **31, 32** Male dorsal and ventral 09-SRNP-56330 **33, 34** female dorsal and ventral 06-SRNP-36773 **35** Male St8 **36** Male genitalia 09-SRNP-56330 **37** Phallus **38** Female genitalia 06-SRNP-36773.

**Male** (Figs 31, 32, 35–37). ***Head*** – Antenna pectinate in basal 4/5, rami moderately long reddish brown, distal fifth simple, shaft gray brown with reddish-brown scales at base, scape with scale tuft gray brown and cream; frons with a mix cream and gray-brown scales; labial palpus upcurved blackish brown with a few scattered cream-colored scales; ocelli absent; vertex gray brown, cream colored laterally; patagium blackish brown with margins cream colored. ***Thorax and abdomen*** – Tegula cream colored at base, a mix of cream and gray-brown scales distally; mesoscutum blackish brown anteriorly, cream and blackish brown posteriorly; mesoscutellum mostly creamy white; thoracic pleuron cream colored to blackish brown; legs mostly reddish brown on outer surfaces, cream colored on inner surfaces. Abdominal dorsum light gray, venter cream colored. ***Wings*** – Dorsal ground color with a mixture of gray-brown, blackish-brown and beige scales; veins lined with gray, especially distally; anal fold and cubitus blackish brown; orbicular spot diffuse blackish brown; reniform spot small, blackish brown; M-line thin, wavy, blackish brown, a wide, vaguely-defined beige band beyond it; PM-line thin, blackish brown, poorly defined; AD-terminal line with blackish-brown spots, fringe gray brown. Ventral surfaces of both wings gray brown. Dorsal hindwing dirty gray brown, lighter near base (Figs 31, 32). ***Male genitalia*** – (Figs 35–37) St8 wide and short, anterior margin simple, posterior margin bearing a simple acute and triangulate process (Fig. 35). Uncus small with a hollow depression in middle, socci thin, long and acute. Valva wide and membranous with saccular margin serrate, heavily sclerotized at base (Fig. 36). Phallus narrow at base, expanding medially, heavily sclerotized at distal third, with dorsal margin serrate, narrowing to tip. Vesica tiny (Fig. 37). **Female** (Figs 33, 34, 38). Antenna filiform, shaft gray brown with a mix of reddish-brown scales; Body color and wing pattern similar to male but wings longer and darker (Figs 33, 34). (WL 16.4–17.0 mm). ***Female genitalia*** (Fig. 38) – Segment 8 forming a heavily sclerotized capsule; anterior apophyses thin and acute; posterior apophyses thin, CB small and round, signum absent; DB short; ostium recessed in St8. Ovipositor lobes acute and setose.

#### Natural history

(Figs 82, 83, 84). 318 caterpillars reared from the western lower and intermediate elevations of the Cordillera Volcanica de Guanacaste (220–1276 m elevation), and the only ACG species of *Dunama* that even marginally occurs in the edge of ACG dry forest. Sector Cacao (n=122), Del Oro (n=8), El Hacha (n=2), Mundo Nuevo (n=169), Pailas (n=16).

Food plants: Arecaceae: *Bactris major* Jacq. (n=2), *Chamaedorea costaricana* Oerst. (n=307), *Geonoma cuneata* (n=8).

#### Parasitoids.

**Braconidae**: Macrocentrinae? *Austrozele*? (n=1); Meteorinae, *Meteorus* Zitani01DHJ05 (n=1). **Tachinidae**: *Lespesia* Wood33DHJ06 (n=6), a species of tachinid parasitoid that it shares with two other species of *Dunama*.

#### Distribution and habitat.

In addition to the rearing records from ACG, *Dunama jessiebancroftae* has been collected in the Peninsula de Nicoya, and the lowland of Central Pacific Costa Rica, from 50 to 1286 m elevation (Fig. 85); all of these extra-ACG sites are also intergrades between rain forest and dry forest, at least before they were largely deforested.

#### Remarks.

This species is homogeneous over its limited range. Nearest neighbor analyses (Fig. 86) suggest that it is the most different from all other *Dunama* in Costa Rica, which suggests the highly unlikely scenario that it was orginally a species of the intergrade of dry forest with rain forest, and then evolutionarily spread into rain forest ecosystems.

### Dunama janecoxae

Chacón sp. n.

urn:lsid:zoobank.org:act:8A6B7BD5-50DD-4231-9B79-AECE8B977669

http://species-id.net/wiki/Dunama_janecoxae

Figs 39–46 , 71–73

#### Type material.

**Holotype** male: 05-SRNP-36040 (Dissected, COI Barcoded), Costa Rica, Prov. Guanacaste, Sector Cacao, Sendero Toma Agua 10.92847, -85.46680, 1140 m, 12 June 2005, Manuel Pereira (INBio). **Paratypes**: 6♂ 3♀. Male: 03-SRNP-3122 (Dissected), Costa Rica, Prov. Guanacaste, Sector Cacao, Sendero Circular, 10.92714, -85.46683, 1185 m, 20 February 2003, Freddy Quesada. Male: 03-SRNP-3121, Costa Rica, Prov. Guanacaste, Sector Cacao, Sendero Circular, 10.92714, -85.46683, 1185 m, 20 March 2003, Freddy Quesada. Female: 05-SRNP-36044 (Dissected, COI Barcoded), Costa Rica, Prov. Guanacaste, Sector Cacao, Sendero Toma Agua 10.92847, -85.46680, 1140 m, 12 March 2005, Manuel Pereira. Female: 03-SRNP-3125, Costa Rica, Prov. Guanacaste, Sector Cacao, Sendero Circular 10.92714, -85.46683, 1185 m, 19 February 2003, Freddy Quesada. Female: 03-SRNP-3223 (Dissected, COI Barcoded), Costa Rica, Prov. Guanacaste, Sector Cacao, Sendero Circular 10.92714, -85.46683, 1185 m, 21 February 2003, Freddy Quesada.

#### Other material examined:

Barcoded: 22 specimens that segregated into four haplotypes with differences from the most common haplotype from Alajuela (14 specimens) of 0.1% for a single haplotype from Alajuela, 1.1% for four specimens from Puntarenas, and 1.4% for three specimens from Cartago. The Cartago and Puntarenas specimens differed by 0.6%. Musem specimens: (14 specimens) 2♂ 1♀ Alajuela, 8♂ Cartago, 2♂ Puntarenas. Dissections: 1♂ Alajuela, 2♂ l♀ Guanacaste, 3♂ l♀ Cartago, 2♂ Puntarenas. **INBio specimens**: Male:INB0004298089(COI Barcoded), Costa Rica, Prov. Puntarenas, Altamira, Cerro Biolley 9.039314, -83.009966, 1700–1800 m, 10 August 2004, R. Delgado. Male: INB0004298088 (COI Barcoded), Costa Rica, Prov. Puntarenas, Altamira, Cerro Biolley 9.039314, -83.009966, 1700–1800 m, 10 August 2004, R. Delgado. Male: INB0004298087(COI Barcoded), Costa Rica, Prov. Puntarenas, Altamira, Cerro Biolley 9.039314, -83.009966, 1700–1800 m, 10 August 2004, R. Delgado. Male: INB0004298086(COI Barcoded), Costa Rica, Prov. Puntarenas, Altamira, Cerro Biolley 9.039314, -83.009966, 1700–1800 m, 10 August 2004, R. Delgado. MaleINB0004298089(COI Barcoded), Costa Rica, Prov. Puntarenas, Altamira, Cerro Biolley 9.039314, -83.009966, 1700–1800 m, 10 August 2004, R. Delgado. Male: INB0004298088 (COI Barcoded),Costa Rica, Prov. Puntarenas, Altamira, Cerro Biolley 9.039314, -83.009966, 1700–1800 m, 10 August 2004, R. Delgado. Male: INB0004298087(COI Barcoded), Costa Rica, Prov. Puntarenas, Altamira, Cerro Biolley 9.039314, -83.009966, 1700–1800 m, 10 August 2004, R. Delgado. Male: INB0004298086(COI Barcoded), Costa Rica, Prov. Puntarenas, Altamira, Cerro Biolley 9.039314, -83.009966, 1700–1800 m, 10 August 2004, R. Delgado. Male: INBIOCRI002010968 (Dissected,COI Barcoded), Costa Rica, Prov. Puntarenas,Est. La Casona, Monteverde 10.298429, -84.792544, 1520 m, 30 January–18 February 1995, K. Martinez, Male: INB0003058436 (Dissected, COI Barcoded), Costa Rica, Prov. Cartago, A.C.L.A.P, Paraiso, Pque Nal Tapanti, Sect La Represa, del Puente del Rio Porras 300 m S. 9.695214, -83.781156, 1660 m, February 2000, L. Chavarria.

#### Etymology.

This species is named in honor of Ms. Jane Cox, mother of Jessie Hill of Philadelphia and Hawaii, and in emphatic recognition of Jessie Hill’s contribution to saving and inventorying the conserved ACG rain forest in which *Dunama janecoxae* breeds.

#### Diagnosis.

St8 wide, short, anterior margin simple, posterior margin densely sclerotized with a rectangular shape lacking any processes; phallus simple, thin at base, heavily sclerotized distally part, with a pair of small triangular projections subopposite on each margin. The medial projection from the costa of the male genitalia is unique among species of *Dunama*.

#### Description.

**Figures 39–46.**
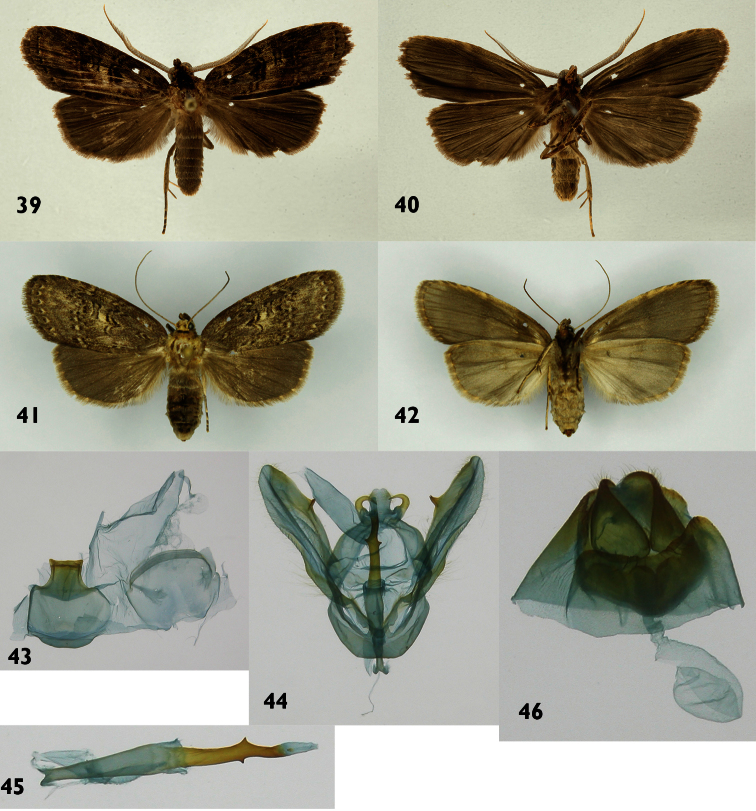
*Dunama janecoxae* **39, 40** Male dorsal and ventral 03-SRNP-36040 **41, 42** Female dorsal and ventral 03-SRNP-3223 **43** Male St8 **44** Male genitalia 03-SRNP-36040 **45** Phallus **46** Female genitalia 03-SRNP-3223.

**Male** (Figs 39, 40, 43–45). ***Head*** – Antenna pectinate in basal 4/5, rami moderately long, reddish brown, distal fifth of shaft simple, gray brown with a mix reddish-brown scales, scape with scale tuft cream; frons with cream scales mixed with blackish-brown scales; labial palpus upcurved, blackish brown with a few scattered cream-colored scales; ocelli absent; vertex blackish brown, cream colored laterally; patagium blackish brown near midline, blackish brown laterally, margins cream colored. ***Thorax and abdomen*** – Tegula cream colored at base, a mix of cream and blackish-brown scales distally; mesoscutum blackish brown anteriorly, cream and blackish brown posteriorly; mesoscutellum mostly creamy white; thoracic pleuron cream colored; legs mostly blackish brown on outer surfaces, cream colored on inner surfaces. Abdominal dorsum light gray, venter cream colored. ***Wings*** – Dorsal ground color a mixture of gray-brown and beige scales; veins lined with gray, especially distally; anal fold and cubitus blackish brown; orbicular spot diffuse blackish brown; reniform spot small, blackish brown; M-line blackish brown, a wide, vaguely-defined beige band beyond it; PM-line thin, blackish brown, poorly defined; subterminal (St) line marks light brown, AD-line with spots ligth brown, fringe gray brown. Dorsal hindwing dirty gray brown, lighter near base. Ventral surfaces of both wings gray brown (Figs 39, 40). (WL 16.1–17.4 mm). ***Male genitalia*** (Figs 43–45) – St8 wide, short, anterior margin simple, posterior margin densely sclerotized with a rectangular shape lacking any process (Fig. 43). Uncus lobule-like with pubescent, thin socii prominently hooked. Valvae with smooth costal margin with a sclerotized, spine-like process near middle. Saccular margin slightly sclerotized at base with a notch in middle (Fig. 44). Phallus thin at base, expanding medially and heavily sclerotized distally, with a pair of small triangular projections, subopposite on each margin; vessica small and without cornuti (Fig. 45). **Female** (Figs 41, 42, 46) – Antenna filiform, shaft cream with a mix of reddish-brown scales; body color and wing pattern similar to male but wings longer (Figs 41, 42). (FW 18.3–18.7 mm). ***Female genitalia*** (Fig. 46) – Segment 8 forming a heavily sclerotized capsule; anterior apophyses acute; posterior apophyses tiny, CB small and rounded, signum absent; DB short; ostium recessed in St8. Ovipositor lobes acute and setose.

#### Natural history

(Figs 71, 72, 73). 61 rearing records from ACG. Sector Cacao (n=35) only. Food plants: Arecaceae,* Chamaedorea costaricana* (n=35) only. *Geonoma* sp. (Arecaceae). Four males RDR00153 reared by Roberto Delgado, Cerro Biolley, Puntarenas Province. Elevational distribution 1090–1185 m.

#### Parasitoids.

**Braconidae**: Microgastrinae, *Parapanteles paradoxus*DHJ03 (n=10), shared with three species of *Tithraustes* Druce (n=4) (Notodontidae: Dioptinae) feeding on the same Arecaceae in the same habitats.

#### Distribution.

*Dunama janecoxae* is the upper elevational species of *Dunama* on the Cordillera Volcanica de Guanacaste, and the eastern slope of Cordillera de Tilaran and Talamanca, occurring from 1090 to 1185 m elevation (Fig. 85).

#### Remarks.

*Dunama janecoxae* seems to offer the classic conundrum of isolated populations in the upper elevations of isolated mountains. Each population has a unique and slightly different barcode (Fig. 86), but the genitalia differ only slightly among populations and far less than that displayed among most other species of *Dunama* in Costa Rica. In as much as we have life history data for only the ACG population, we elect to leave these mountaintop populations as one species, even though their morphological and barcode differences are of the same degree as other ACG sympatric/parapatric pairs of species with distinct but similar barcodes (e.g., Janzen et al. 2005; *Neoxeniades luda* (Hewitson) and *Neoxeniades pluviasilva* Burns (Burns et al. 2007); four sympatric/parapatric species of *Perichares* Scudder (Burns et al. 2008)). Additional material will be needed to determine the extent of separation of these different montane populations.

### Dunama biosise

Chacón sp. n.

urn:lsid:zoobank.org:act:3EACC77B-1111-45B5-A55D-A66FB60B054A

http://species-id.net/wiki/Dunama_biosise

Figs 47–54

#### Type material.

**Holotype** male:INB0003558870 (Dissected), Costa Rica, Prov. Puntarenas,Sirena, Corcovado Nat. Pk., Osa Penin 8.479267, -83.588565, 0–100 m, 19–27 March 1981, D.H. Janzen & W. Hallwachs (INBio). **Paratypes**: 3♂ 1♀. Male: INBIOCRI000494661(COI Barcoded), Costa Rica, Prov. Puntarenas, P. N. Corcovado, Est. Sirena 8.479267, -83.588565, 0–100 m, December 1991, G. Fonseca. Male: INBIOCRI000674591, Costa Rica, Prov. Puntarenas,P. N. Corcovado, Est. Sirena 8.479267, -83.588565, 0–100 m, March 1991, G. Fonseca. Male: INBIOCRI002583632, Costa Rica, Prov. Puntarenas,Sirena, Corcovado Nat. Pk., Osa Penin. 8.479267, -83.588565, 0–100 m, 10–12 August 1980, D.H. Janzen & W. Hallwachs. Female: INBIOCRI002527271 (Dissected), Costa Rica, Prov. Puntarenas,Sirena, Corcovado Nat. Pk., Osa Penin. 8.479267, -83.588565, 0–100 m, 15–25 March 1981, D.H. Janzen & W. Hallwachs.

#### Other material examined.

Museum specimens: 4♂ Puntarenas, Dissections: 2♂ 1♀ Puntarenas. **INBio specimens**: Male: INBIOCRI002583652, Costa Rica, Prov. Puntarenas,Sirena, Corcovado Nat. Pk., Osa Penin. 8.479267, -83.588565, 0–100 m, 15–16 August 1980, D.H. Janzen & W. Hallwachs. Male: INBIOCRI002582980 (Dissected), Costa Rica, Prov. Puntarenas,Sirena, Corcovado Nat. Pk., Osa Penin. 8.479267, -83.588565, 0-100 m, 19-27 March 1981, D.H. Janzen & W. Hallwachs. Male: INBIOCRI002582985, Costa Rica, Prov. Puntarenas, Sirena, Corcovado Nat. Pk., Osa Penin. 8.479267, -83.588565, 0–100 m, 19–27 March 1981, D.H. Janzen & W. Hallwachs. Male: INBIOCRI002582981, Costa Rica, Prov. Puntarenas, Sirena, Corcovado Nat. Pk., Osa Penin. 8.479267, -83.588565, 0–100 m, 19–27 March 1981, D.H. Janzen & W. Hallwachs.

#### Etymology.

*Dunama biosise* is named in honor of BIOSIS, the non-profit publishing company, the sale of which generated the JRS Biodiversity Foundation (http://www.jrsbdf.org ), which in turn supports biodiversity information management for conservation in many places, including INBio and ACG.

#### Diagnosis.

St8 wide, short, anterior margin simple, posterior margin sclerotized with a pair of forceps-like processes, a small and sclerotized triangular projection at the base of each process. Phallus with subbasal, unsclerotized expansion, distal half narrow and sclerotized.

#### Description.

**Figures 47–54.**
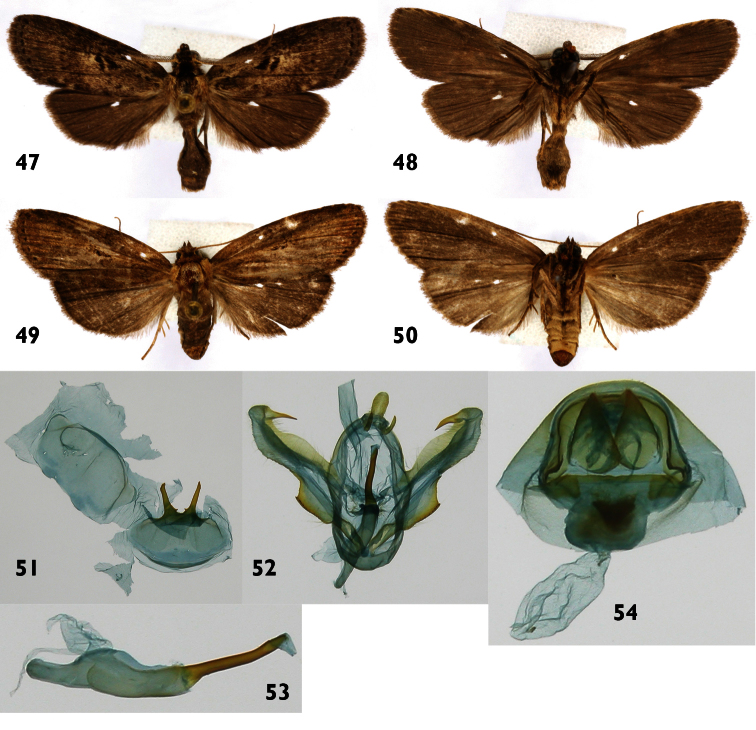
*Dunama biosi*se **47, 48** Male dorsal and ventral INB0003558870 **49, 50** Female dorsal and ventral INBIOCRI002527271 **51** Male St8 **52** Male genitalia INB0003558870 **53** Phallus **54** Female genitalia INBIOCRI002527271.

**Male** (Figs 47, 48, 51–53).***Head*** – Antenna pectinate in basal 4/5, rami moderately long, reddish brown, distal fifth simple, shaft cream colored, scape with scale tuft reddish brown and cream colored; frons with cream scales mixed with reddish-brown scales; labial palpus upcurved reddish brown with a few scattered cream-colored scales; ocelli absent; vertex reddish brown, cream colored laterally; patagium blackish brown near the midline, blackish brown laterally, margins cream colored. ***Thorax and abdomen*** – Tegula cream colored at base, a mix of cream and reddish-brown scales distally; mesoscutum blackish brown anteriorly, cream and blackish brown posteriorly; mesoscutellum mostly creamy white; thoracic pleuron cream colored; legs mostly blackish brown on outer surfaces, cream-colored on inner surfaces. Abdominal dorsum light gray, venter cream colored. ***Wings*** – Dorsal ground color a mixture of gray-brown and beige scales; veins lined with gray, especially distally; anal fold and cubitus ligth brown; orbicular spot diffuse blackish brown; M-line diffuse blackish brown; AD-line with ligth brown spots, fringe gray brown. Dorsal hindwing dirty gray brown, lighter near base. Ventral surfaces of both wings gray brown (Figs 47, 48). (WL 11.7–12.8). ***Male genitalia*** – (Figs 51–53). Tg8 oval, posterior margin narrroly sclerotized; St8 wide, short, anterior margin simple, posterior margin sclerotized with a pair of forceps-like processes, a small and sclerotized triangular projection at base of each process (Fig. 51). Uncus lobule-like and elongate with thin, pubescent socci, up-curved. Valva with costal margin smooth, bearing an apical spine-like projection, long and sclerotized; saccular margin serrate and heavily sclerotized at base, with laminate-like structure (Fig. 52). Phallus with subbasal unsclerotized expansion, distal half narrow and sclerotized. Vesica small, without cornuti (Fig. 53). **Female** (Figs 49, 50, 54). Antenna filiform, shaft cream colored; body color and wing pattern similar to male but wings longer (Figs 49, 50). (WL 13.9 mm). ***Female genitalia*** – (Fig. 54) segment 8 forming a heavily sclerotized capsule; anterior apophyses acute; posterior apophyses tiny, CB small and round, signum absent; DB short; ostium recessed in St8. Ovipositor lobes acute and slightly pubescent.

#### Natural history.

Unknown, except that it is a moth of Costa Rica’s lowland Pacific coast rain forest and both sexes can be captured at light at night.

#### Distribution.

*Dunama biosise* has been collected from 0 to 100 m elevation in the Osa Peninsula, Area de Conservacion Osa (Fig. 85).

#### Remarks.

The single sample submitted for barcode analysis produced only a 349 base pair DNA barcode instead of the hoped-for 658 base pairs (Fig. 86). However, its barcode differences, and its distinctive genitalia, as well as the ecosystem it occupies, all indicate that it is a species distinct from the other known Costa Rican *Dunama*.

### Dunama indereci

Chacón sp. n.

urn:lsid:zoobank.org:act:B5159750-A549-4F2F-932C-521D9FD1FC12

http://species-id.net/wiki/Dunama_indereci

Figs 55–62 , 63–64

#### Type material.

**Holotype** male:INB0004251736 **(**Dissected, COI Barcoded),Costa Rica, Prov. Alajuela, San Ramon, Est. Biol. Villa Blanca 10.201361, -84.485101, 1115 m, September 2009, R. Rojas (Reared). **Paratypes**: 1♂ 2♀. Male: INB0004251737 (COI Barcoded), Costa Rica, Prov. Alajuela, San Ramon, Est. Biol. Villa Blanca 10.201361, -84.485101, 1115 m, September 2009, R. Rojas (Reared). Female: INB0004251729 (COI Barcoded), Costa Rica, Prov. Alajuela,San Ramon, Est. Biol. Villa Blanca 10.201361, -84.485101, 1115 m, September 2009, R. Rojas (Reared). Female: INB0004251730 (Dissected, COI Barcoded), Costa Rica, Prov. Alajuela,San Ramon, Est. Biol. Villa Blanca 10.201361, -84.485101, 1115 m, September 2009, R. Rojas (Reared).

#### Other material examined.

Barcoded: 5 Alajuela (Fig. 86). Musem specimens: (5 specimens) 3♂, 2♀ Alajuela. Dissections: 3♂, 3♀Alajuela. **INBio specimens**: Male: **INB0004251734**. (COI Barcoded) Costa Rica. Prov. Alajuela. San Ramon, Est. Biol. Villa Blanca 10.201361, -84.485101, 1115 m, September 2009, R. Rojas (Reared). Male: **INB0004251733**. Dissected. (COI Barcoded) Costa Rica. Prov. Alajuela. San Ramon, Est. Biol. Villa Blanca 10.201361, -84.485101, 1115 m, September 2009, R. Rojas (Reared). Male: **INB0004251731**. Dissected. (COI Barcoded) Costa Rica. Prov. Alajuela. San Ramon, Est. Biol. Villa Blanca 10.201361, -84.485101, 1115 m, September 2009, R. Rojas (Reared). Female: **INB0004251732**. Dissected. (COI Barcoded) Costa Rica. Prov. Alajuela. San Ramón, Est. Biol. Villa Blanca. 10.201361, -84.485101, 1115 m, September 2009, R. Rojas (Reared). Female: **INB0004251735**.Dissected. (COI Barcoded) Costa Rica. Prov. Alajuela. San Ramon, Est. Biol. Villa Blanca 10.201361, -84.485101, 1115 m, September 2009, R. Rojas (Reared).

#### Etymology.

*Dunama indereci* is named in honor of the International Development Research Centre (IDRC) of Canada in recognition of their support of information management and DNA barcode taxonomy at INBio for conservation, and particularly for its support of the International Barcode of Life Project (iBOL initiated by the Biodiversity Institute of Ontario at the University of Guelph, Canada).

#### Diagnosis.

St8 wide, short, anterior margin simple, posterior margin sclerotized and serrate with four processes, lateral processes shorter than the medial processes; phallus thin, unsclerotized and expanding subbasally, distal half sclerotized with a small trifurcate, spine-like projection on basal ventral margin, on the dorsal margin a tiny spine-like projection; tip with two, large spine-like projections. Vesica very small, without cornuti.

#### Description.

**Figures 55–62.**
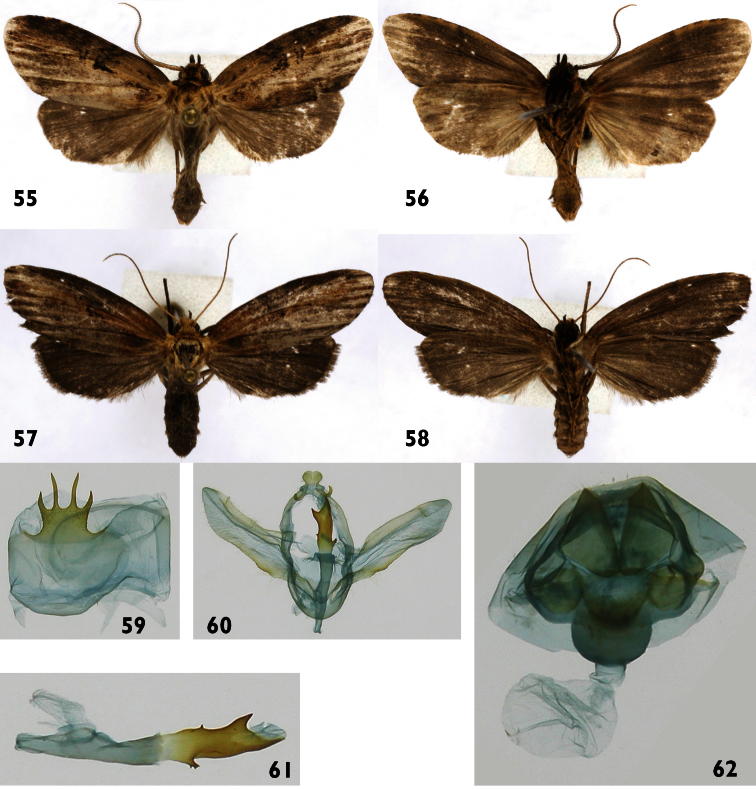
*Dunama indereci* **55, 56** Male dorsal and ventral INB0004251736 **57, 58** Female dorsal and ventral INB0004251730 **59** Male St8 **60** Male genitalia INB0004251736 **61** Phallus **62** Female genitalia INB0004251730.

**Male** (Figs 55, 56, 59–61). ***Head*** – Antenna pectinate in basal 4/5, rami moderately long and reddish brown, distal fifth simple, shaft cream colored, scape with scale tuft blackish brown and cream colored; frons with cream scales mixed with reddish-brown scales; labial palpus upcurved reddish brown with a few scattered cream-colored scales; ocelli absent; vertex reddish brown, cream colored laterally; patagium blackish brown near de midline, blackish brown laterally, margins cream colored. ***Thorax and abdomen*** – tegula cream colored at base, a mix of cream and reddish-brown scales distally; mesoscutum blackish brown anteriorly, cream and blackish brown posteriorly; mesoscutellum mostly creamy white; thoracic pleuron cream colored; legs mostly reddish brown on outer surfaces, cream colored on inner surfaces. Abdominal dorsum light gray, venter cream colored. ***Wings*** – Dorsal ground color a mixture of gray-brown and beige scales; veins lined with gray, especially distally; anal fold and cubitus light brown; orbicular spot blackish brown; reniform spot diffuse, blackish brown; fringe gray brown. Dorsal hindwing gray brown. Ventral surfaces of both wings gray brown (Figs 55, 56). (WL 11.8–12.9 mm). ***Male genitalia*** (Figs 59–61) – St8 wide, short, anterior margin simple, posterior margin sclerotized and serrate with four processes, lateral processes shorter than medial ones (Fig. 59). Uncus wide, pubescent, lobulate-like, with a hollow depression in middle. Socii thin up-curved and hook-like. Valva sclerotized along margins and membranous centrally, distal margin simple, saccular margin serrate with a small spine-like projection in ventral surface near apex (Fig. 60). Phallus thin, unsclerotized and expanding subbasally, distal half sclerotized with a small trifurcate, spine-like projection on basal ventral margin, on dorsal margin a tiny spine-like projection; tip with two, large spine-like projections. Vesica very small, without cornuti (Fig. 61). **Female** (Figs 57, 58, 62). Antenna filiform with yellow-cream shaft; body color and wing pattern similar to male but wings longer (Figs 57, 58). (FW 13.0–14.3 mm). ***Female genitalia*** (Fig. 62) – Segment 8 forming a heavily sclerotized capsule; anterior apophyses acute; posterior apophyses tiny, CB evident and rounded, signum absent; DB short; ostium recessed in St8. Ovipositor lobes pubescent, with acute apex.

#### Natural history

**Figures 63–70.**
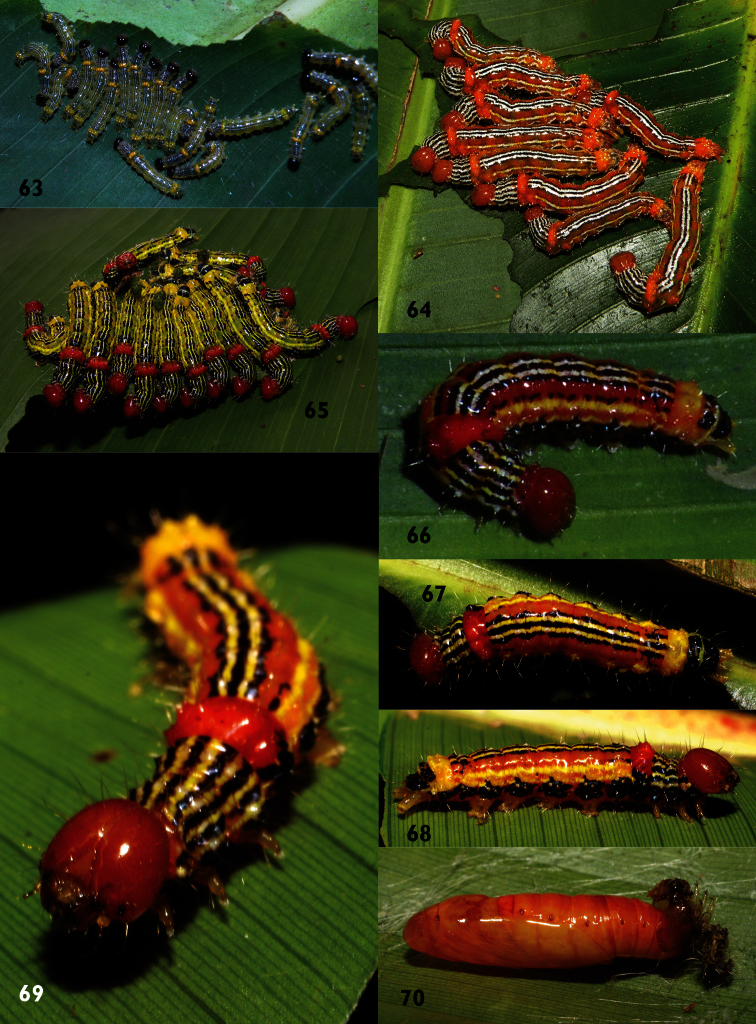
Instars of *Dunama indereci* and *Dunama jessiebarronae* on food plants *Musa* and *Heliconia* **63** Intermediate instar *Dunama indereci* **64** Last instar *Dunama indereci* **65** Penultimate instar *Dunama jessiebarronae* **66–69** Last instar *Dunama jessiebarronae* 06-SRNP-40360 **70** Pupa of *Dunama jessiebarronae* 06-SRNP-40401.

(Figs 63, 64). Food plant: Heliconiaceae: *Heliconia latispatha* Benth., Villa Blanca (9). No parasitoids were reared from this small sample.

**Figures 71–84.**
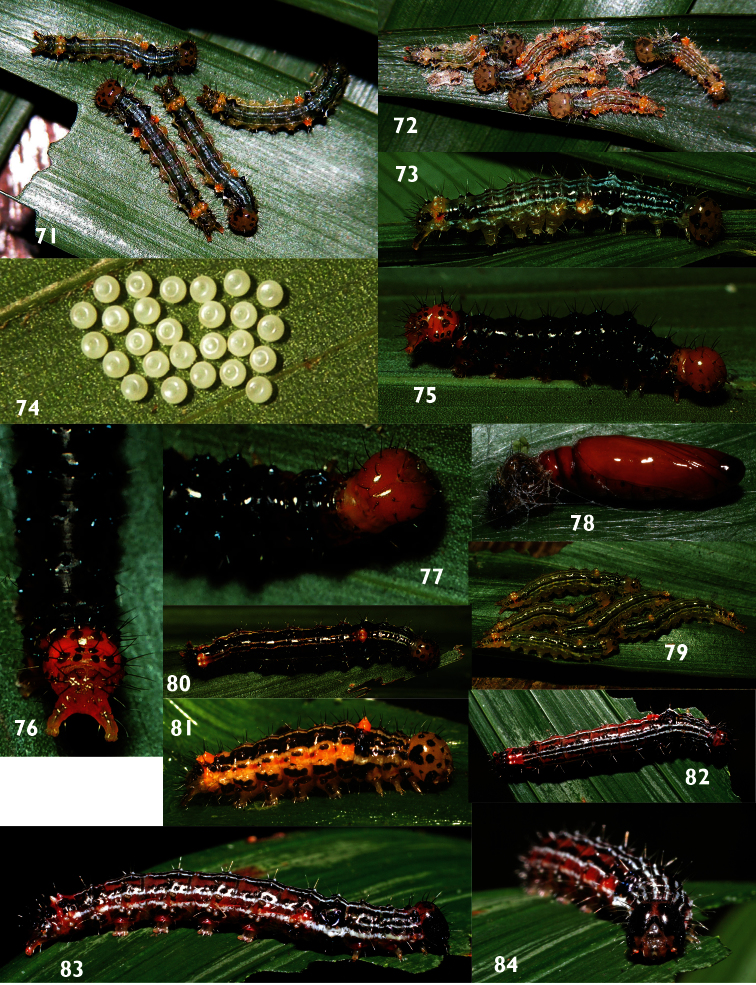
Instars of *Dunama*on food plant (Arecaceae) **71–73** Last instar *Dunama janecoxae* 03-SRNP-3122 **74** Eggs of *Dunama jessiehillae* **75–77** Last instar of *Dunama janewaldronae* 02-SRNP-6497 **78** Pupa of *Dunama janewaldronae* 09-SRNP-40001 **79** Penultimate instar *Dunama jessiehillae* 00-SRNP-11377 **80** Last instar *Dunama jessiehillae* 99-SRNP-4114 **81** Prepupa of *Dunama jessiehillae* 06-SRNP-4940 **82–84** Last instar *Dunama jessiebancroftae* 09-SRNP-56324.

#### Distribution and habitat.

**Figure 85.**
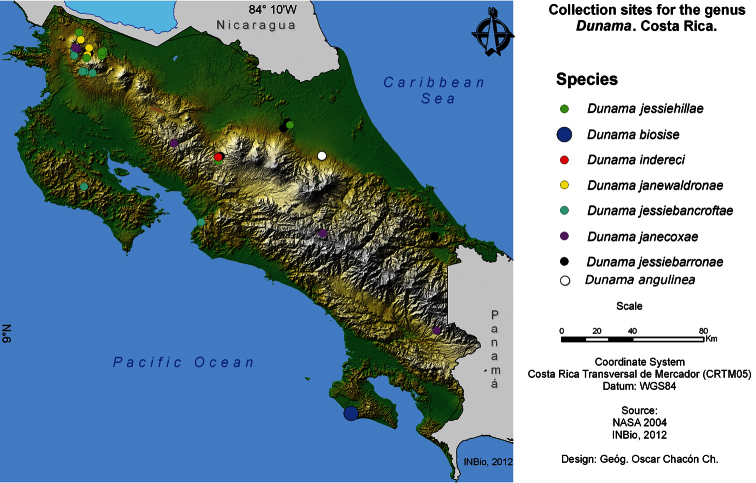
Map of Costa Rican collection sites for the eight species of *Dunama* (Notodontidae) discussed here.

*Dunama indereci* has been collected only in Villa Blanca, in San Ramon, Alajuela province, at 1115 m elevation, in a montane pass between Costa Rica’s Cordillera de Tilaran and Volcanica Central (Fig. 85).

#### Remarks.

**Figure 86.**
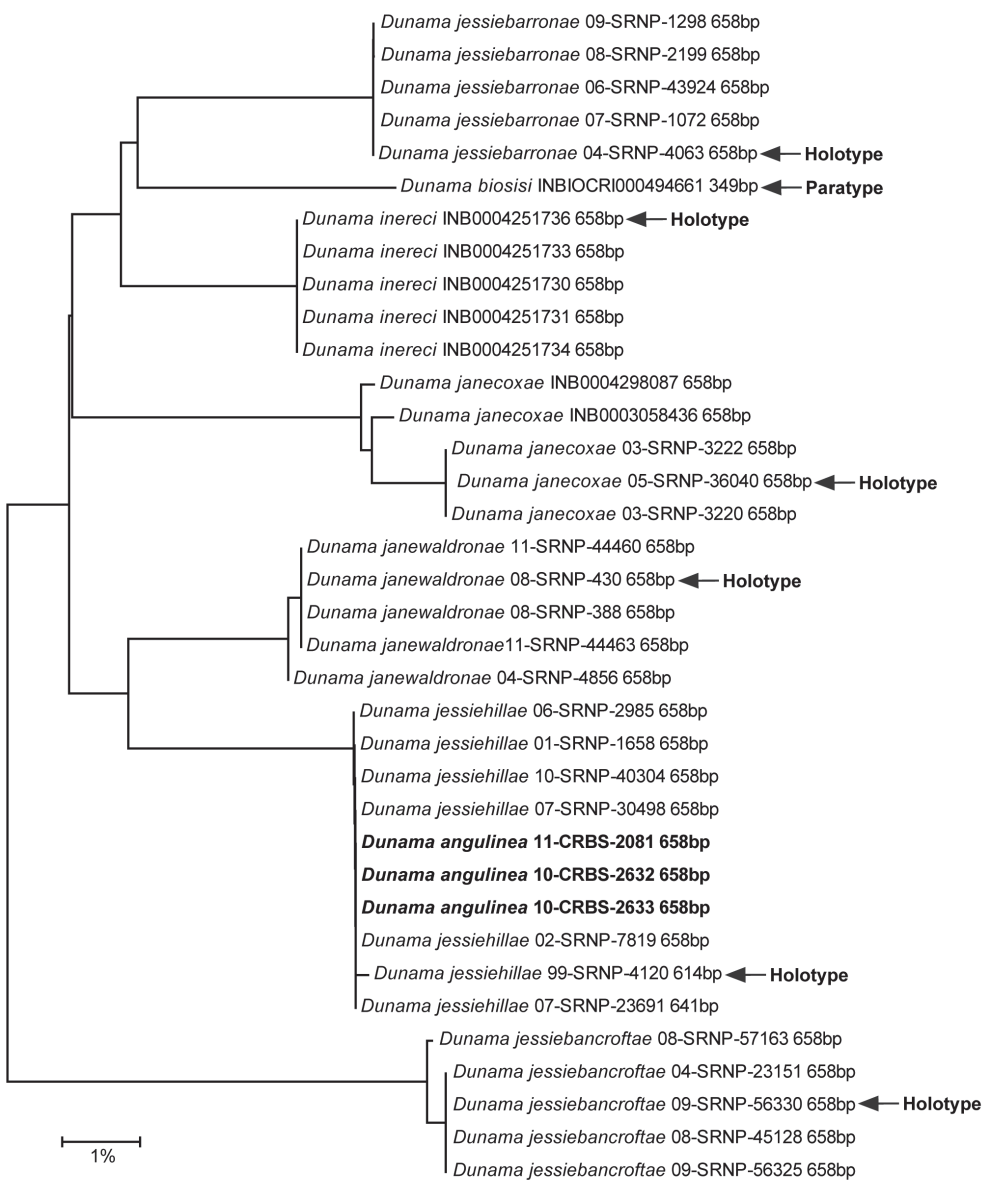
The *Dunama* species from Costa Rica in an NJ barcoding tree. Sample sizes are restricted to a haphazardly selected set of five specimens for each species. Many specimens were reared from wildcaught caterpillars and further information on each can be found at Janzen and Hallwachs (2012).

This species feeds exclusively on *Heliconia latispatha*. One barcode haplotype was recored in the population from Villa Blanca (Fig. 86).

## Supplementary Material

XML Treatment for Dunama

XML Treatment for Dunama angulinea

XML Treatment for Dunama jessiehillae

XML Treatment for Dunama jessiebarronae

XML Treatment for Dunama janewaldronae

XML Treatment for Dunama jessiebancroftae

XML Treatment for Dunama janecoxae

XML Treatment for Dunama biosise

XML Treatment for Dunama indereci
